# Direct Optical Patterning of Quantum Dots: One Strategy, Different Chemical Processes

**DOI:** 10.3390/nano13132008

**Published:** 2023-07-05

**Authors:** Francesco Antolini

**Affiliations:** Fusion and Technologies for Nuclear Safety and Security Department, Physical Technology for Safety and Health Division, ENEA C.R. Frascati, Via E. Fermi 45, 00044 Frascati, Italy; francesco.antolini@enea.it

**Keywords:** semiconductor quantum dots, polymers, ligands, siloxanes, thiol-ene, quantum dots stability, quantum dots dispersion, patterning, cross linkers

## Abstract

Patterning, stability, and dispersion of the semiconductor quantum dots (scQDs) are three issues strictly interconnected for successful device manufacturing. Recently, several authors adopted direct optical patterning (DOP) as a step forward in photolithography to position the scQDs in a selected area. However, the chemistry behind the stability, dispersion, and patterning has to be carefully integrated to obtain a functional commercial device. This review describes different chemical strategies suitable to stabilize the scQDs both at a single level and as an ensemble. Special attention is paid to those strategies compatible with direct optical patterning (DOP). With the same purpose, the scQDs’ dispersion in a matrix was described in terms of the scQD surface ligands’ interactions with the matrix itself. The chemical processes behind the DOP are illustrated and discussed for five different approaches, all together considering stability, dispersion, and the patterning itself of the scQDs.

## 1. Introduction

This review aims to have a critical overview of direct optical patterning (DOP) of the semiconductor quantum dots (scQDs), as closely related to their function, which is in turn linked to their stability and homogeneity. For real exploitation of the scQDs in a commercial device, this material should have three main characteristics, i.e., (i) stability in the conditions of usage, (ii) homogeneity of distribution within the device to guarantee uniform performances, and, for some applications, like in displays, (iii) the possibility to be patterned. These three characteristics, however, cannot be taken alone, because they are interconnected. Indeed, the development of the chemical processes that underlies the stability and dispersion has to be compatible with the adopted patterning technology.

The patterning strategies of scQDs can be divided into three main groups: photolithography [1], contact printing [2], and ink-jet printing [3]. Several works describe all of these techniques and here we want to highlight the aspect of interrelation, which is often overlooked, between the patterning methodology and the scQDs’ functionality, i.e., stability and dispersion. Some recent developments in patterning are exploring the direct use of light as a tool for patterning as in the “classical” photolithography, but to control the position of the object of interest, the scQDs, without the use of masks and repeated steps of layer curing/etching typical of the photolithographic process. If, on one side, the direct optical patterning (DOP) of scQDs simplifies the process of patterning, on the other side this shifts on the material side the critical issues of the scQDs’ dispersion, protection, and patterning itself. Indeed, the role of light is to block the scQDs in a specific position by creating a network that sticks them, by changing the scQDs solubility or by growing them directly.

This review is, then, organized by presenting briefly the state of the art of the scQDs’ materials (Section 1), the role of the ligands in the dispersion of the scQDs in a matrix (Section 2), the main critical issue regarding the scQDs’ stability involving oxygen moisture and temperature (Section 3), the main strategies to overcome the degradation issues at a single and collective level (Section 4), and finally how specific chemical processes developed in the previous sections interplay with the DOP (Section 5).

### 1.1. The Semiconductor Quantum Dots

Semiconductor quantum dots [4] (scQDs) are among the most studied and utilized nanomaterials because their compositional and morphological tunability modulates their optoelectronic properties that can be adapted for different applications. On the other side, the relatively easy synthesis in a colloidal state further contributes to their real-world use.

There are two main approaches for semiconductor QD fabrication [5]: the physical methods and the wet-chemical methods.

The physical methods include the molecular beam epitaxy (MBE) and the metal-organic chemical vapor deposition (MOCVD) that allow the preparation of thin layers of semiconductor QDs or deposit them over a wafer.

The chemical methods involve the synthesis in the liquid phase (organic solvent or water) at relatively high temperatures (100–350 °C) in the presence of precursors and surfactants. The controlled combination of the precursors, their ratio, the presence of surface ligands, the reaction temperature, and the duration of the heat treatment determines the stoichiometry, size, and shape of the scQDs. The atomic species forming the scQDs belong to the elements from II–VI, III–V, IV–VI, and IV groups, and include the metal halide perovskites (CsPbX_3_ X = I, Br or Cl) [6,7,8].

The possibility to control the electro-optical properties of the scQDs through all of these parameters without changing the whole chemistry of the system is a key advantage of this class of materials and opens many research paths [9]. However, finding the right combination of them is not an easy task, especially in the case of core/shell systems (see the next sections). For example, a careful selection of precursors and ligands is strictly connected with the temperature of nucleation and growth of the scQDs, and together their optical properties. For example, if the growth and nucleation processes are not well distinguished final scQDs are not monodispersed (monodispersion means that all the scQDs have the same size) and, hence, the FWHM of the preparation is broad. In the same way, the high crystalline quality of the core ensures a high quality of performance [10].

The modulation of the density of states, i.e., the tuning of the scQDs’ electro-optical properties, is another technological aspect that is deeply studied. One strategy is to modify the shape of the nanocrystals, as illustrated in Section 2.1, while another research direction is the doping of scQDs as reported by Mocatta et al. [11].

Another critical aspect of the scQDs’ synthesis that influences their application in thin films is the role of the ligands that mediate the interaction with the substrate and with themselves. The next sections (Section 2.1 and Section 2.2) show the effect of the ligands in solid state and the role of the inorganic ligands which helps the scQDs’ close packing and electron transfer between the scQDs [12].

### 1.2. The Quantum Size Effect and Its Role in the Modulation of the Electro-Optical Properties of the scQDs

**Figure 1:**
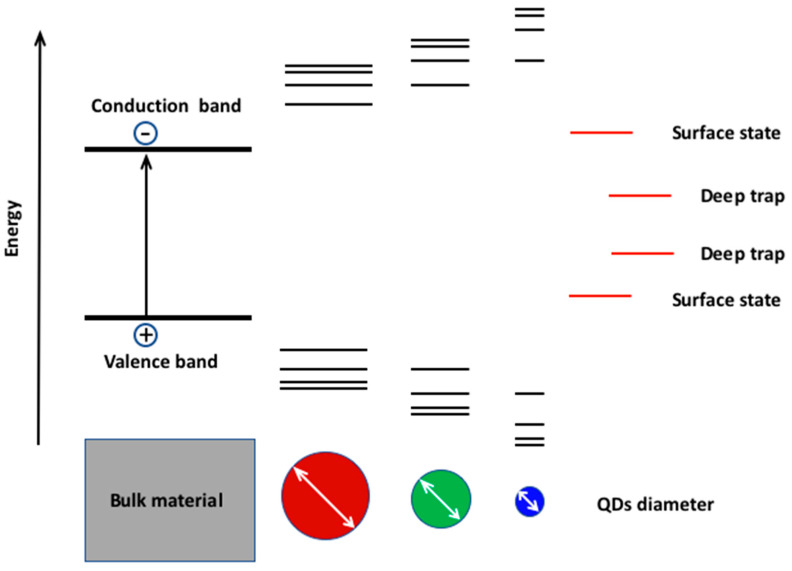
The valence band and the conduction band of the semiconductor bulk material become quantized (black bars) when the size of the QD (white arrow) becomes smaller than the Bohr diameter. The defect surface states and deep traps (red bars) are due to surface defects and crystal defects.

The importance of this class of nanomaterials lies in the possibility of modulating their optoelectronic properties by their size, composition, and architecture. The modulation of the optical properties by size is the so-called quantum size effect [9]. This effect takes place when the size of the semiconductor becomes smaller than the wavefunction of the exciton (the electron and hole pair formed by Coulomb interaction) [13], typically below 10 nm, depending upon the type of material [9]. In this condition, the bandgap of the semiconductor material becomes quantized and the effective bandgap increases, decreasing the particle size and modifying the absorption and emission properties (Figure 1).

In such a way, the bandgap of the scQDs can be tuned from different energy levels from the ultraviolet to infrared range. The bandgap fine-tuning enables the specific emission wavelength, the size uniformity of the emitting cores causes the narrow emission, while the absence of crystal defects mainly at the surface (Figure 1) ensures a high photoluminescent quantum yield (number of emitted photons per absorbed photons) and also contributes to narrow emission.

The wavelength tunability (color selection), the narrow emission (color purity), and the photoluminescent quantum yield (brightness) are characteristics of paramount importance for the application on a device. The improvement of the knowledge of the scQDs showed that the source of the problems are the defects states from surfaces of the scQDs (surface traps and deep traps) that perturb the band gap structure, changing the emission wavelength and lowering the luminescent quantum yield.

### 1.3. The Core@shell Systems

The solution for the stabilization of the scQDs structure arrived from the introduction of the core@shell systems [14,15,16]. In this configuration, the surface defects are fixed by growing an inorganic shell over the scQD core. The shell role is two-fold: the passivation of the surface defects and the localization of the exciton into the core [5]. The growth of another type of material over the scQD should be chosen carefully because the shell material should crystallize over the core without introducing any mechanical (crystallographic) stress, which means more surface defects.

Another crucial point for the realization of the desired core/shell system is the reaction methodology. It is possible to summarize three different methods of growth of the core/shell systems; namely, the two (multiple)-steps synthesis, the one-pot synthesis, and the SILAR (successive ion layer adsorption and reaction). The two-steps reaction is the most used approach because it allows the removal of the reaction byproducts after each reaction. Cao et al. [17], for example, prepared a multiple core/shell/shell system like CdSe/CdS/ZnS with this approach. First, they synthesize the core that is used, after the purification, as a reagent for the synthesis of the shell. The CdSe/CdS is then purified and utilized for the growth of the ZnS final layer. In this case, the metal precursors are CdO, Cd(OAc)_2_, and Zn(OAc)_2_, while the selenium and sulfur are added slowly to the reaction mixture. The slow addition of the chalcogenides prevents the nucleation of the shell material. The SILAR methodology forecasts the formation of the shell layer-by-layer. Each layer is realized by two different injections in the reaction vessel of the cationic and anionic precursors [18].

The one-pot synthesis approach consists of the formation of the scQDs using a core with a gradient shell. W.K. Bae et al. [19] introduced this approach, suggesting that the growth of this type of material, indicated as Cd_1−x_Zn_x_Se_1−y_S_y_, is due to different reactivity of the reagents (metal and chalcogenides) mixed in the same vessel when reacting at the same temperature (the reaction is carried out at 300 °C). All of these reaction approaches are widely used by researchers that developed many particular variations, always to improve the photoluminescent quantum yield (PLQY), the FWHM, and the scQDs’ stability. 

Cao et al. [17] sorted out a CdSe/CdS/ZnS scQD with an electroluminescence efficiency six orders of magnitude higher than the standard ruthenium complex. That can be used for an electrochemical immunoassay for the development of QD-LEDs. Hanifi et al. [20] set up a method of core/shell synthesis that produces CdSe/CdS scQDs having a PLQY approaching 1. These scQDs with this high PLQY are developed for applications in the photovoltaic field.

Other examples of core-shell systems are the so-called giant QDs. They are systems with a very high shell/core volume ratio (shell thickness higher than 1.5 nm) and the absorption is dominated by the shell [21,22]. These systems are particularly advantageous because they exhibit a high distance between the absorption and emission maxima (Stokes-shift) that improves the efficiency of the optoelectronic devices where these systems are included.

A further example of this band engineering for the optimization of optoelectronic properties is the modulation of the shell shape, like the dot in rods [23,24] and nanoplatelets [25]. Both engineered structures show a high Stokes-shift between the absorption band and emission band that ensures the absence of the reabsorption of the emitted light [26]. Both types of structures were described in this section because the ligands play a pivotal role in their preparation.

## 2. scQD Dispersion

### 2.1. The Surface Ligands

Another important structure characterizing the scQDs are the surface ligands [27,28]. Even if they are not part of the crystalline structure of the scQDs, they mediate the interaction of the nanocrystals with the surrounding environment.

**Figure 2:**
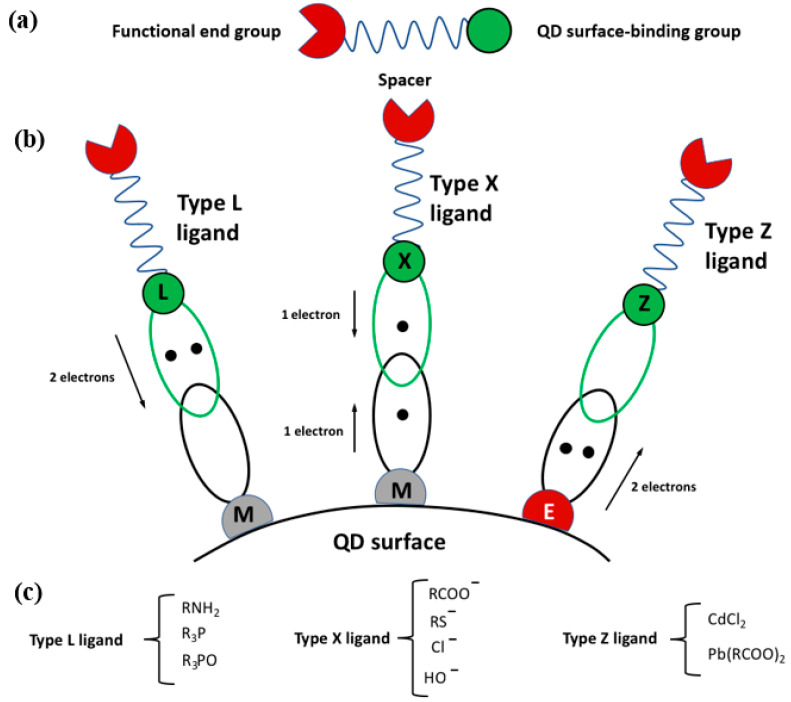
(**a**) Block scheme of the three sections of which the ligand is formed: the functional end group that interacts with the environment, the spacer, and the scQD surface-binding group that interacts with the scQD surface; (**b**) the L, X-types of ligand (green circle) can share with their molecular orbitals (green ellipse) 2 or 1 electron with the molecular orbitals of the metal atoms. The Z-type ligand can accept two electrons from the scQD surface. In general, the electron-poor atoms are metal (gray atom labeled as “M”) while the electron-rich is, chalcogenide (red atom labeled as “E”); (**c**) examples of molecules belonging to the different classes of ligands.

At the molecular level, the ligand can be thought of as formed of three main blocks (Figure 2a): the surface binding group, the spacer, and the functional end group. The binding group is responsible for the bond of the ligand at the metal or the chalcogen, pnictogen, or halogen non-metal atom of the scQDs, depending upon the type of binding group as described below. The spacer distances the binding group from the end group, and its length, in particular, can modulate the energy transfer between the close particles, influencing the electron transfer [29] or the solubility [30]. The end group interacts directly with the surroundings so that it can bind other molecules [31,32], if suitably adapted, or can modulate more effectively the dispersion within the solvent [33].

The binding of the ligands over the scQD surface can be qualitatively understood in terms of a Lewis acid/base concept. Following this rule, they can be classified into three groups [34] (Figure 2b): the ligand can interact with the scQD surface by donating two electrons (Lewis base), and, in this case, it belongs to the L-type group, like amines, phosphines, and phosphine oxides (Figure 2c). The ligands with one electron donor belong to the X-type group and they require on the scQD surface one atom with an electron to form a covalent bond. The typical ligands are carboxylates, thiolates, and hydroxyls (Figure 2c). The Z-type ligands are molecules that can accept two electrons (Lewis acid) from another atom over the scQD surface. The typical ligands are cadmium and lead cations (Figure 2c).

The surface ligands play different roles at the scQD surface: (i) they control the growth of the scQDs during their synthesis; (ii) they stabilize the surface dangling bonds as the inorganic shell, modifying the scQDs’ electronic structures; and (iii) they mediate their interaction with the solvent or with the material in which they are in contact [27].

The most important effect of the ligand during the synthesis is the prevention of aggregation of the nanocrystals during the growth. 

The role of the surface ligand is therefore to act as a “screen” versus the external environment controlling the nucleation and the growth of the nanocrystal itself [35]. Following the La Mer model, a nanocrystal is formed when the monomers (atoms necessary to form the nanocrystals) reach the supersaturation concentration [36]. In this condition takes place the so-called nucleation burst (sudden growth of many nuclei). When the concentration of the precursors drops, the nucleation stops and the nuclei start to grow. The QD growth is governed by the minimization of the surface energy; however, the nanocrystals facets have not the same surface energy so that the final shape depends also from the surface energy of each facet (function of the broken bonds exposed in each facet). This rule becomes particularly critical when the ligands are present, because they can have different affinity of the ligand versus the different facets of the growing nanocrystals. The ligands, indeed, can cap specific sites, slowing the growth rate of the passivated facets producing rod or plate like symmetries (chemical poisoning effect). The nature of the ligand is equally important and a small difference produces different results. An example of the shape control was achieved by using a mixture of octadecyl and hexyl phosphonic acids that stimulates the growth of CdS rods around the CdSe seeds, as shown by Carbone et al. [23].

Another ligand property is their propensity to form closely packed layers (soft templating effect). The close packing of the ligand tails ensures a stable assembling, hindering the growth on the crystallographic plane covered by the ligand [37,38]. This assembly is also controlled by the temperature that can disorganize the close packing of the tails. An example of such behavior was studied by Li et al. [38], where they obtained CdSe disks blocking the growth along one axis by using a polar ligand (fatty carboxylates). This growth is also modulated by the temperature, when increased above the temperature limit of the fatty acid close packing.

The binding of the ligand stabilizes the scQDs from an electronic point of view, by the energy levels of the scQDs fixing the surface defects, i.e., eliminating the surface traps and modulating the scQDs’ band gaps [39].

For the scQDs’ application, however, the main characteristic of the ligand is its interaction with the surroundings, and this action is played mainly by the end group, often in synergy with the spacer.

### 2.2. The Ligands and the Surrounding Environment of the scQDs

The ligands mediate the compatibility of the scQDs within the matrix. The best way to enhance the dispersion is to prepare an scQD with a ligand compatible with the host matrix [40]. The dispersion of the scQDs in a matrix is a particularly important factor for the scQDs’ application, especially in a solid state like in a film, because the aggregation phenomena quench the electro-optical properties of the scQDs [41,42,43], nullifying all of the efforts made to obtain nanocrystals with excellent optical properties.

An elegant example of the function of the ligand as a “tool” to optimize the dispersion and, hence, the optical properties of the scQDs is found in the work reported by Lesnyak’s group, where the CdSe nanoplatelets (NPLs) were functionalized with a ligand bearing a modified end group, improving the NPLs’ dispersion [44] in a polymer. 

**Figure 3:**
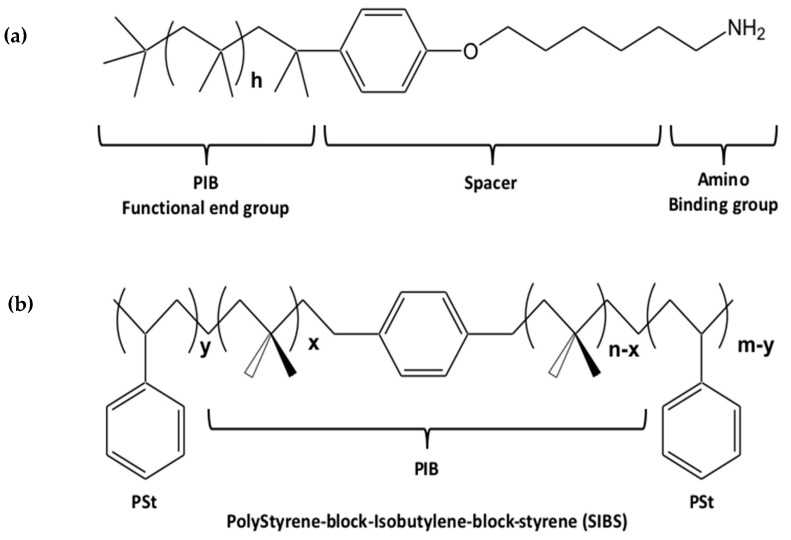
(**a**) The scQDs ligand was engineered with the amino-terminal group connected with the PIB chain through the spacer benzene ring–oxygen and six carbon atoms. The number of isobutylene units (h) forming the PIB backbone of the ligand is about 18; (**b**) chemical formula of the block copolymer Polystyrene-block-isobutylene-block-styrene (SIBS) that is the matrix embedding the scQDs.

In this report, the authors faced the typical problem of the scQDs’ application; that is, their dispersion at high concentration in a matrix that has specific characteristics. The high dispersion of the NPLs within the polyisobutylene (PIB) polymer was obtained by functionalizing the NPLs with a ligand bearing, as the functional end group, a short tail as in PIB (Figure 3a), and that has the same chemical nature of the PIB polymer. The same chemical nature of the ligand and the polymer (Figure 3b) ensures the complete miscibility of the QDs in the polymer. 

The tests of dispersion and stability which were carried out, comparing the spreading of the NPLs in three types of polymers (namely: the poly(lauryl methacrylate) (PLMA), the PIB, and the PIB block copolymer (SIBS)) showed the formation of very high transparent films [44], which indicates an optimal dispersion.

Despite a huge amount of work conducted on the study of the organic ligands, it is worth describing also the use of the inorganic ligands. Indeed, they can replace the organic ligands to improve the charge transport between the scQDs [12,45].

Talapin’s group recently showed how the native organic ligand, typically the oleic acid, can be replaced by metal-inorganic salts [12] to obtain intensely luminescent all-inorganic nanocrystals (ILANs). The metal-inorganic salts they used for surface passivation of the scQDs include the metal cations of Cd^2+^, Zn^2+^, Pb^2+^, and In^3+^ with anions like NO_3_^−^, BF_4_^−^ e triftalate (OTf^−^).

**Figure 4:**
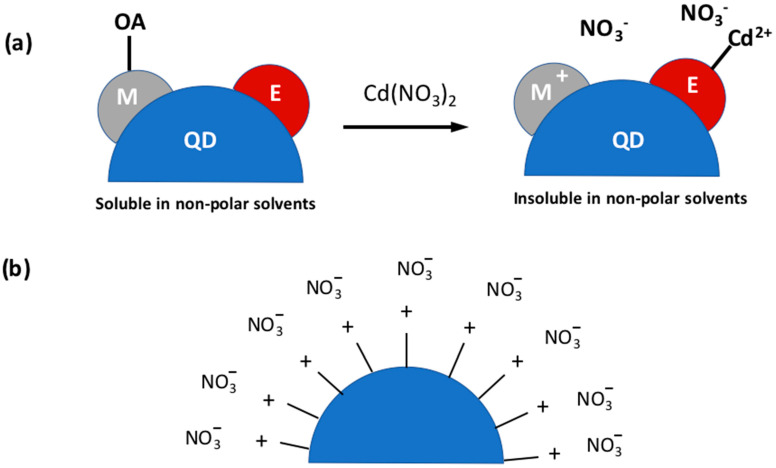
(**a**) Scheme of the dual effect of the inorganic ligand Cd(NO_3_)_2_: the stripping of the oleic acid (OA) and the binding to the non-metal atom (E). The change in ligand induces the change in solubility; (**b**) the anions just balance the charge near the cations that bind the electron-poor metal site forming a kind of cloud around the QD: a bound cation surrounded by a cloud of anions.

The role of the metal cations is (Figure 4a) (i) to remove the native organic ligands, and (ii) to bind the non-metal atom, on the scQD surface. In terms of the Lewis acid-base concept, the ligand metal cation, a Lewis acid, coordinates the electron-rich chalcogenide atom, a Lewis base, at the scQD surface. On the other side, the anion acts as a charge balancer rather than a coordinating agent (Figure 4b). The main effect of this ligand exchange is the variation of the scQDs’ solubility (dispersion) of the scQDs in solvents. Indeed, the solubility of the scQDs in non-polar solvents (hexane, toluene, etc.) switches to solubility in polar solvents (DMF, NMF, DMSO, etc.). 

What is important to highlight is that the procedure of the ligand exchange proposed by Talapin’s group leaves the optical characteristics of the scQDs almost unchanged with a slight decrease in the PLQY [12]; that is a typical drawback of the inorganic ligand exchange.

## 3. QD Stability

### 3.1. The Effect of Oxygen and Moisture

Answering the question about the stability of the scQDs under ambient conditions in combination with light will help to adopt the necessary countermeasures to improve the life of any device equipped with this material.

For ambient conditions, this means the effect of the oxygen, moisture, and working temperature on the functional characteristics of the scQDs, namely: the emission wavelength, the luminescence broadening, the photoluminescent quantum yield (PLQY) [46], and the PL intermittency (blinking) [47,48]. What is clear is that the roles of oxygen, moisture, and temperature, and their combination with light, are crucial, as highlighted in different reports testing the stability of the QDs used for display [49], solar cells [50], and fluorescent probes for biology [51]. On the other side, the studies on scQD stability in the presence of oxygen and water are often contradictory to each other for several reasons, such as different quality of the scQDs prepared with different procedures, non-ideal structural and optical quality, and different testing conditions.

Only recently, the group led by Peng clarified the role of oxygen [52] and water [53] by studying systematically their effect on a well-defined system, the CdSe/CdS core/shell scQDs, in defined experimental conditions in terms of atmosphere (only oxygen, only water, or their defined combination) and different phases at single scQD level or as an ensemble of scQDs in thin film and solution.

The study of the role of oxygen [52] shows that, at the functional level, this molecule maintains the bright state both of the single scQDs and when the scQDs are embedded together in a film (photoactivation). When the oxygen is removed, for example with argon, the scQDs enter a dim state (low emission and small PL shift). The proposed mechanism is that during the photoexcitation, there is the possibility that the scQDs form a trion (two electrons and one hole in the scQDs) bringing the scQDs in the dim charged state (off state). The “bright” state is restored with the presence of oxygen that accept one electron forming the superoxide radical (^•^O_2_^−^). The oxygen reduction returns the scQDs to charge neutrality restoring the scQDs’ optical properties. Another interesting conclusion of this work is that the high quality of the shell avoiding any hole and electron surface traps does not allow any effect of corrosion of the scQDs by the oxygen (the redox potential of the oxygen is quite different from the core/shell scQDs). On the other side, the redox potential of the oxygen should be able to oxidize the surface of bare CdSe scQDs (no core/shell scQDs), especially under photoexcitation producing CdO, SeO_2_, and CdSeO_x_ [54,55]. The authors conclude that pure oxygen helps to maintain the photophysical properties, and it is not responsible for photo-corrosion of high-quality core/shell nanocrystals. The controversial results found in the literature on the role of oxygen may be due to the non-ideal quality of the prepared core/shell scQDs allowing the corrosion, as reported for the bare CdSe QDs.

**Figure 5:**
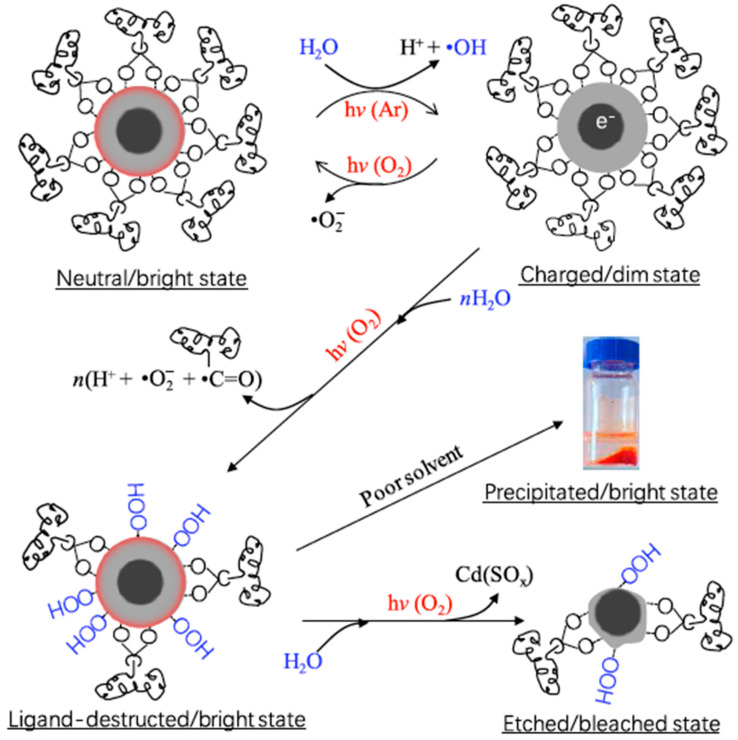
Scheme of the corrosion process initiated by water ionization of the scQDs that can bring to a partial degradation of the ligands, then to corrosion of the shell with the final loss of the photophysical properties of the scQDs (Reprinted with permission from [53]. Copyright © 2021 American Chemical Society).

Peng’s group studied also the role of water combined with oxygen, showing that this combination is responsible for the corrosion and the loss of the photophysical properties of the scQDs [53]. The complete “story” of the water and oxygen interaction with the scQDs starts with the “ionization by water and deionization by oxygen” step, as reported in Figure 5. In this first step, the excited nanocrystal is negatively ionized (reduced) by water that dissociates, producing a very reactive specie, the hydroxyl radical (^•^OH) and protons (H^+^). The negatively charged scQDs are now in the dim state, but the presence of the oxygen, as shown before, brings the scQDs to a neutral state, restoring its bright state and producing as a byproduct the superoxide ion (equilibrium between neutral/bright state and charged/dim state; Figure 5). The presence of the radical species, especially the hydroxyl radical (^•^OH) formed under the continuous presence of water and irradiation, brings to an acidic pH and a carboxylate ligand detachment from the scQDs’ surfaces. This phenomenon causes the poor solubility of the QDs in the solvent and the precipitation of the bright scQDs (precipitated/bright state, ligand-destructed/bright state; Figure 5). The loss of the surface ligand exposes the inorganic shell to the formation of surface traps bringing further chemical decomposition even of the shell of the scQDs (photo-corrosion) with irreversible loss of their photophysical properties (etched/bleached state; Figure 5). 

It is worth mentioning that when the scQDs are confined in an area with no access to water and oxygen, like in the display applications (the QD’s film is isolated from the environment), the balance between the brightening and dimming states reaches an equilibrium and the decomposition cannot go ahead.

**Figure 6:**
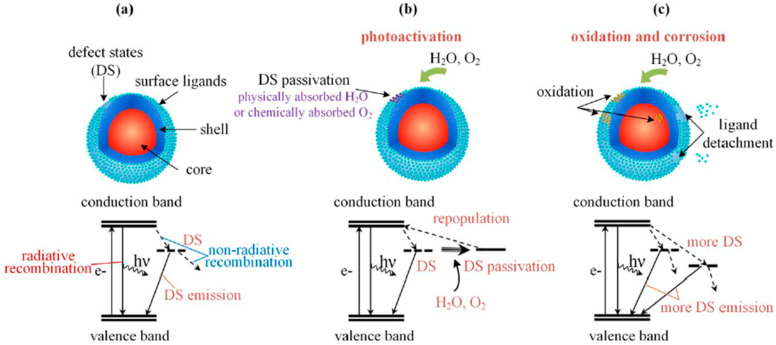
Scheme of the photophysical processes involving the scQDs (**a**) when they are excited with light; (**b**) under the presence of a limited amount of oxygen and water; or (**c**) in the continuous presence of them. (Reprinted with permission from [46]).

At the photophysical level, the effect of the oxygen and water in the scQDs can be resumed as reported by Yang et al. in Figure 6 [46]. In this view, the core/shell scQDs, when prepared, can have just a few defect states (DS) (depending upon the optimization of the chemical synthesis), meaning that the scQDs have a determined PL emission, FWHM, and PLQY, function of size and composition (valence and conductive band) (Figure 6a). The number of defect states and the presence of non-radiative recombination (NRR) paths contribute to the values of the three parameters above.

In the presence of a limited amount of oxygen and water (scQDs protected from the external conditions), the oxygen fixes the defect states repopulating the conduction band, so that the photophysical properties (PL emission wavelength, PLQY, and FWHM) are restored or even improved (photoactivation) [56] (Figure 6b).

If the presence of oxygen and moisture is continuous, the oxidation and corrosion processes rise, meaning that the defect states increase, worsening the photophysical properties of the scQDs (Figure 6c).

### 3.2. The Effect of the Temperature

The thermal stability of the scQDs is another crucial factor influencing their practical applications [49]. In general, the effect of the temperature increase on the QDs’ function is that a loss of PL intensity [57,58] takes place. This loss can become irreversible if the temperature rise causes the loss of ligands or shell degradation. Despite its importance for a real-world application, the mechanism of the PL quenching induced by the temperature is still not clear. What is commonly accepted is that the PL thermal quenching is associated with the activation of a surface state that traps a charge (whether positive or negative is still under debate) [57,59,60] with subsequent non-radiative recombination processes. In any case, whatever the mechanism is, the scQD surface plays a central role in PL quenching induced by the temperature.

**Figure 7:**
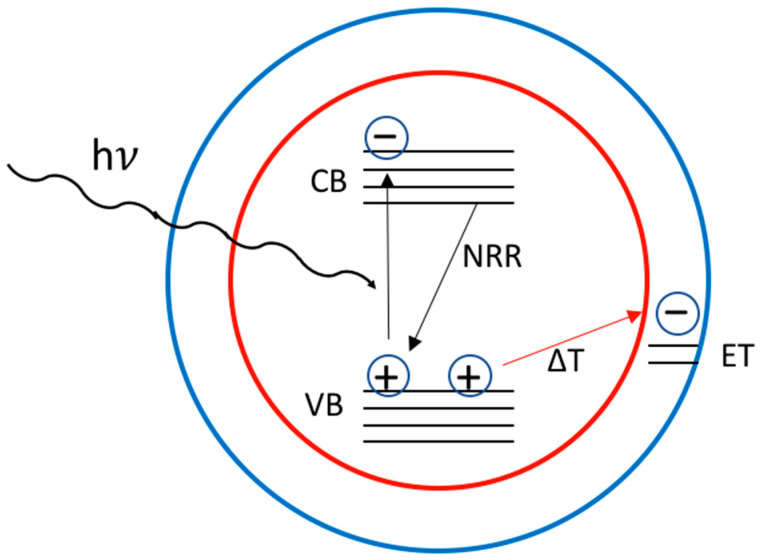
Mechanism of switching off of the scQD induced by temperature: the temperature (ΔT) stimulates the formation of the electron trap (ET) on the scQD surface (the blue circle indicates the inorganic shell) and induces the transition of one electron from the valence band (VB) of the scQD core (red circle) to the trap level on the surface shell. If the light stimulates the transition of one electron into the conducting band (CB), it decades into the valence band (VB) through non-radiative recombination (NRR).

Cai et al. [57] studied the effect of the temperature in the range RT to 100 °C in solution (toluene) in core/shell scQDs like CdSe/ZnSe and CdTe/CdSe, depending also on different ligands. This report suggests that the main process taking place is the ionization of the core with the formation of a trion. The main phenomenon occurring is that a valence band electron shifts to a surface state (ET in Figure 7) having similar energy by the effect of the temperature. In this condition, the core is charged positively while the shell is negative, and therefore the scQD remains neutral. If there is the absorption of a photon, the scQD core becomes a positive “trion” in which two holes are in the valence band of the core and one electron is in the conducting band of the core itself. In this configuration, the scQD is in the “dark” condition and the electron within the core relaxes in a non-radiative way (Figure 7). The electron confined in the ET on the surface can decay through non-radiative recombination [59], and if the trap state is reversible, even the photophysical properties of the scQDs can be restored [61].

Other studies [61] that were carried out at higher temperatures (up to 200 °C) for different structures such as core/shell/shell, dot in rod both in solution (octadecene), and in solid state (embedded in polymers) reveal that the effect of photoluminescence quenching is faster in solution than in the solid state. This effect is expected because, in a hot solvent, the loss of surface ligands is easier and can be permanent, creating permanent traps.

The mechanism of the photoluminescence quenching is always the creation/activation of a surface state that traps the charge carrier. The role of the surface on the thermal stability of the photoluminescence has been observed even for the giant scQDs under high flux of light at 100 °C, in which the thicker inorganic shell separates the emissive core from the nanocrystal surface, increasing the temperature stability of the scQDs [60].

## 4. Stabilization of the QDs at a Single and Collective Level

The scQDs’ deterioration, i.e., the loss of the ligands and atoms from their structure, can be prevented by adopting two different strategies: (i) the encapsulation of each single scQD and (ii) the encapsulation of the scQDs embedding them within a matrix.

In the former, the main idea is to overcoat the single QD with a material that “freezes” its structure and hence its properties so that they can be incorporated into the final device. In the second approach, they are dispersed in a covalent network that does not protect the single scQDs, but all of the scQDs. The encapsulation methodologies include mainly silica, polymers, and metal-organic frameworks (MOFs) [62,63]; however, we have focused our attention on silica and polymers because these two matrices are more flexible in terms of scQDs dispersion, have the possibility to be patterned, and are available on an industrial scale. 

### 4.1. Single QD Encapsulation

In general, for the single scQD stabilization, two different materials are used, namely: silica (SiO_2_) [64,65] or polymers [66].

The silica is a good encapsulation material for the scQDs because is transparent, stable against chemicals, and is a good barrier for oxygen and moisture. Another crucial factor of its widespread coupled with nanomaterials concerns the use of mild conditions of synthesis (room temperature and relatively simple precursors and solvents) that can be adapted, depending on the different types of nanomaterial [63].

**Figure 8:**
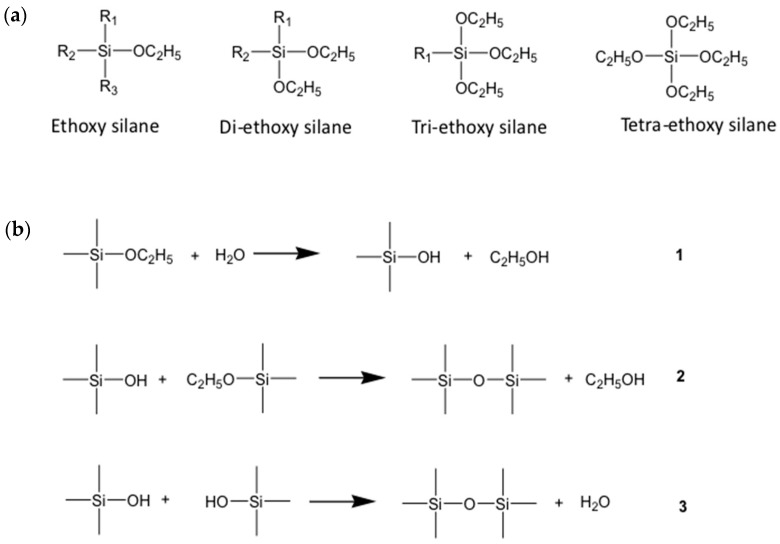
(**a**) Silane precursors of siloxanes and (**b**) the condensation reactions bringing to the formation of the Si-O-Si bonds.

From the chemical point of view, the silica coating can be prepared by starting from mono or di, tri, or tetra-functional silanes. In these compounds, each atom of silicon is bound to one, two, three, or four reactive alkoxy groups that, in acidic or basic conditions in the presence of water, condense and form the Si-O-Si bonds (Figure 8) [63,67]. Depending upon the type of monomer and the reaction conditions, it is possible to achieve different types of architectures from random networks, ladders, partial cages, and perfect cages.

The most important side effect of the silica encapsulation of scQDs is the significant quenching of the scQDs’ PLQY after the encapsulation reaction. However, this drawback can be minimized by changing the polymerization conditions, modulating the scQDs’ architecture and shell thickness [65], or introducing other reaction methodologies like the microemulsion approach [68].

Zhou et al. [64], for example, prepared silica-coated CdSe/ZnS scQDs exchanging the native oleic acid ligand with a silane ligand, namely the mercaptopropyl trimethoxy silane (MTS), then they formed the silica layer by adding the tetraethyl orthosilicate (TEOS) and ammonium hydroxide for the condensation reaction. The silica coating (about 10 nm) was then further functionalized with methylsilane to make the QDs/Silica hydrophobic. These scQDs were then used to prepare silicone disks for testing their resistance upon water immersion. These disks showed a constant PLQY of about 77% for 15 days.

Other authors used the microemulsion method and shell optimization to improve the PLQY of the overcoated scQDs [65,69]. On one side, the microemulsion method allows the native ligands to be maintained over the QDs, minimizing the surface defects. On the other side, a thicker shell or a specific scQD architecture confine tightly the electron–hole pair in the QD core, far from the surface defects.

**Figure 9:**
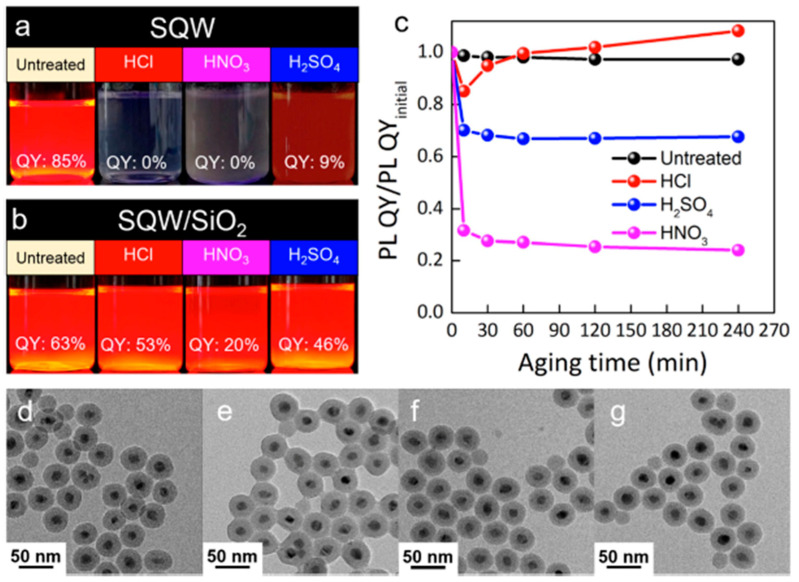
Photograph under UV light of (**a**) unprotected SQW in acidic conditions; (**b**) silica overcoated SQW in acidic conditions; (**c**) evaluation of the PLQY of the SQW protected with silica in acidic conditions (the un-protected samples are practically not luminescent); TEM images of the SQW/SiO_2_ (**d**) without acid etching (shell thickness 17 nm) and SQW/SiO_2_ etched for 48 h with (**e**) HCl, (**f**) HNO_3_ (**g**) H2SO_4_ (Reprinted with permission from [65]).

Wang et al. [65], for example, improved the PLQY of the scQDs by introducing a core/shell/shell quantum well (SQW) architecture as a stronger emissive material. They found that after the silica overcoating of the CdS/CdSe/CdS SQW, the PLQY decreased from 85% (native) to 68% (overcoated). Goryacheva et al. [69] showed that by optimizing the microemulsion conditions and the shell thickness in a core/shell CdSe/CdS QDs, the PLQY decreased from 80% (native) to 70% (overcoated). The role of the silica coating in the scQDs’ optical properties preservation was shown by Wang et al. [65], who exposed the SQW to harsh acidic conditions. In these conditions, the PLQY of the unprotected material was 0% in almost all cases, while the silica-overcoated SQWs maintained or even improved their optical performances (Figure 9).

Ko et al. [66] proposed an encapsulation with polymers that is based on a specially designed ligand whose role is (i) to ensure the distribution of the scQDs within an organic matrix and (ii) to form a network of covalent bonds around every single scQD protecting it from the degradation.

**Figure 10:**
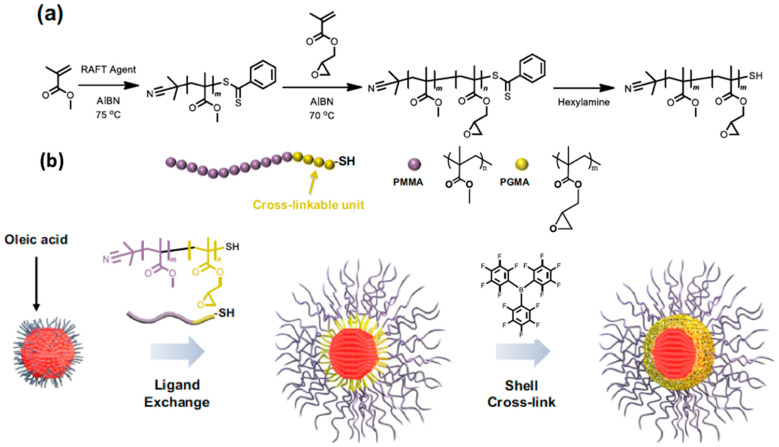
(**a**) Chemical steps of the synthesis of the smart ligand bearing PMMA tail, the epoxy cross-linkable units, and the thiol end group; ((**b**)-top) scheme of the ligand PMMA tail, a spacer of cross-linkable units (PGMA) and thiol binding group; ((**b**)-bottom) steps of the overcoating process of the scQDs from ligand exchange to cross-linked shell around the scQD (Reproduced with permission from [66]).

The methyl methacrylate (MMA) monomers form a PMMA tail of the ligand, which is necessary to ensure the correct dispersion of the scQDs on the PMMA matrix. Near the scQDs’ binding group, the MMA units are substituted by a cross-linkable unit formed by a short sequence of polyglycidyl methacrylate (PGMA). This sequence bears the epoxydic group that opens forming a covalent bond with another epoxydic group [70]. Then, the scQDs’ binding group consists of a thiol that is a strong surface binding group (Figure 10a,b). This ligand is exchanged with the native ligand used for the scQDs’ synthesis and then the new ligand, bearing the cross-linking units of PGMA, was activated to form a network around the scQD to protect it from the degradation (Figure 10b).

The thermal treatment at 100 °C in toluene of the native scQDs capped with oleic acid and the ones overcoated with cross-linked ligand showed that the encapsulated QDs maintain 85% of their initial PLQY value, while the native scQDs retain only 40% of their initial value.

Similar results are obtained when the scQDs are exposed to air or hydrogen peroxide.

The stability was also studied in a solid state (composite formed by PMMA and scQDs) after the treatment for two hours at 100 °C. In these conditions, the native scQDs dispersed in PMMA showed in the time-resolved PL spectra of a significative presence in its lifetime curve of a fast non-radiative recombination component that is practically absent in the overcoated scQDs. It is worth pointing out that the dispersion of the PMMA-b-GMA-QDs is homogeneous throughout the film, as observed both with TEM and AFM, because the ligand was optimized with a tail homogeneous with the PMMA matrix, while the native scQDs were covered with oleic acid that aggregates within the PMMA.

### 4.2. Collective QD Encapsulation

#### 4.2.1. The Thiol-Ene Network

**Figure 11:**
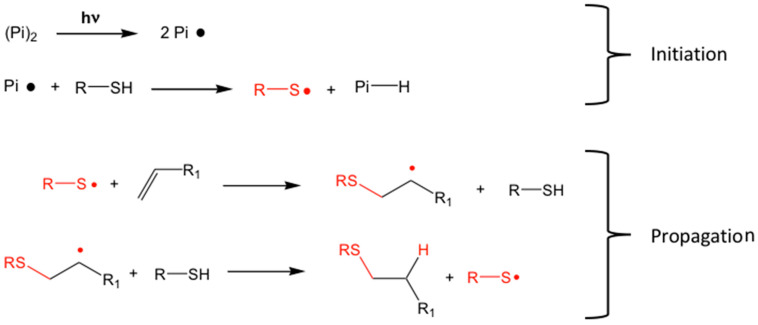
Mechanism of thiol addition to a C-C double bond mediated by a radical Photo-initiator (Pi) stimulated by light, which starts the production of the thiyl radical (R-S) that then binds the double bond (propagation).

A common way of collective encapsulation of the scQDs within a matrix is to use molecules forming a network through the thiol-ene chemistry [71,72]. The term thiol-ene refers to a reaction where a thiol molecule is added to a carbon–carbon double bond (“ene” bond), regardless of the reaction mechanism, that can be either radical [73] or nucleophilic [74]. In the following pages, the radical mechanism will be described because it is stimulated by light (Figure 11). The initiation involves the activation of the thiol with a photo-initiator (Pi) through light. The formed thiyl radical (R-S •) reacts with a carbon–carbon double bond that then activates a second molecule of thiol, propagating the reaction. A possible termination process is the radical–radical coupling process.

It is worth highlighting that this reaction is simple to perform, is not sensitive to water and oxygen, and the reagents are readily available. The addition of the thiol to the carbon–carbon double bond is quite general in the sense that the olefinic bond can be substituted or not. In the same way, virtually, any type of thiol can be used for the addition. The last important property is that this reaction is quite rapid and complete in a few seconds, and there is no need to remove any byproduct.

With the help of this chemical process, several papers have been published involving the encapsulation of the scQDs.

**Figure 12:**
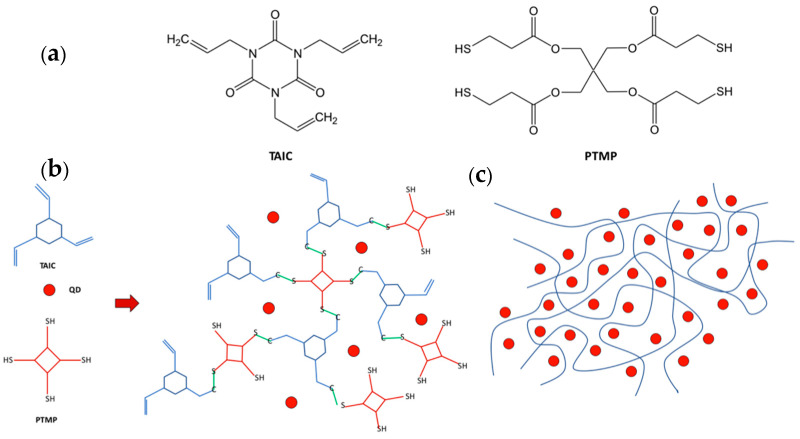
(**a**) Reagents for the thiolene click reaction utilized to encapsulate the scQDs; (**b**) the four branched thiol PTMP and TAIC form chemical bonds creating the network around the scQDs; (**c**) the schematic view of the final polymer network around the scQDs. The reader should imagine a 3D network surrounding the scQDs represented by the red circles.

Smith et al. [75] use a triene (1,3,5,-triallyl-1,3,5-triazine-2,4,6(1H,3H,5H)-trione) (acronym TAIC), a three-branched allyl molecule, and a four branched thiol (pentaerythritol tetrakis(3-mercaptopropionate) (acronym PTMP) to encapsulate the QDs in a matrix of well-distributed scQDs. The thiol-ene reaction between the TAIC and PTMP allows the formation of a network encapsulating the scQDs (Figure 12).

Unfortunately, the mixture of the triene and the four-branched thiol includes also a urethane-based component, so it is impossible to speculate more about the chemical properties of the system. The optical properties of the scQDs are maintained because the scQDs were not subjected to further processing, like ligand exchange or other treatments, while the use of buthylamine as a ligand of the scQDs avoids any interference of the oleic acid double bond with the thiol-ene reaction [76].

#### 4.2.2. The Polymers

The collective encapsulation with polymers was widely used [62]; however, the direct mixing of polymers and scQDs, even if it is relatively simple, suffers from the issue of scQD aggregation. When there is a chemical interaction between the scQDs and the polymer, the encapsulation strategy is more effective. 

**Figure 13:**
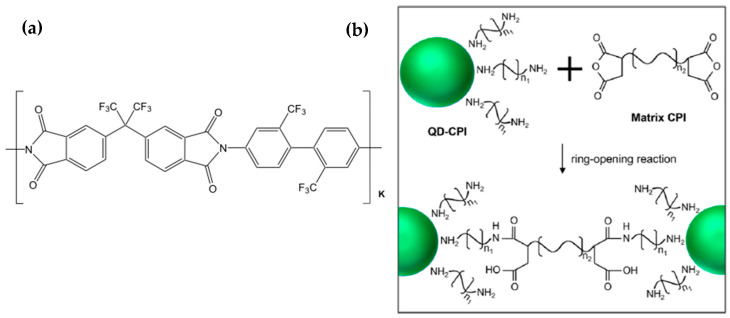
(**a**) Chemical structure of the monomeric unit forming the CPI; (**b**) scheme of the reaction of cross-linking of the CPI ends, the phthalic anhydride, and the amino group of the CPI ligands bound on to the scQD surface (Reproduced with permission from [77]. Copyright © 2022 American Chemical Society).

Heo et al. [77] reported this type of interaction that adopted a colorless polyimide (CPI) as a polymeric matrix embedding and interacting covalently with the scQDs. In this work, the authors exploit the chemical, thermal, and mechanical properties of the transparent polyimide to stabilize the scQDs in ambient conditions (presence of moisture oxygen, and temperature). Indeed, the presence of the fluorine-containing groups in the CPI [78] prevents water penetration within the film (Figure 13a).

In this work, a core/shell scQD is grafted with a diamine-terminated polyimide ligand. This ligand is then covalently bound to the CPI polymeric matrix through its terminating ends of phthalic anhydride that reacts with the amino group of the ligand itself (Figure 13b) [78].

The same chemical structure of the ligands and the polymer (ligands contain CPI oligomers) ensures good dispersion of scQDs within the CPI and more stability against moisture and oxygen, due to the CPI properties. 

In this approach, however, the polymer does not form any network stabilizing and protecting the scQDs. This difficulty can be circumvented not only by selecting a suitable polymer, but also by forming an effective encapsulation network of the polymer around the scQDs. Lesnyak’s group gave an interesting example of how these two obstacles, dispersion and stability, can be circumvented [79].

**Figure 14:**
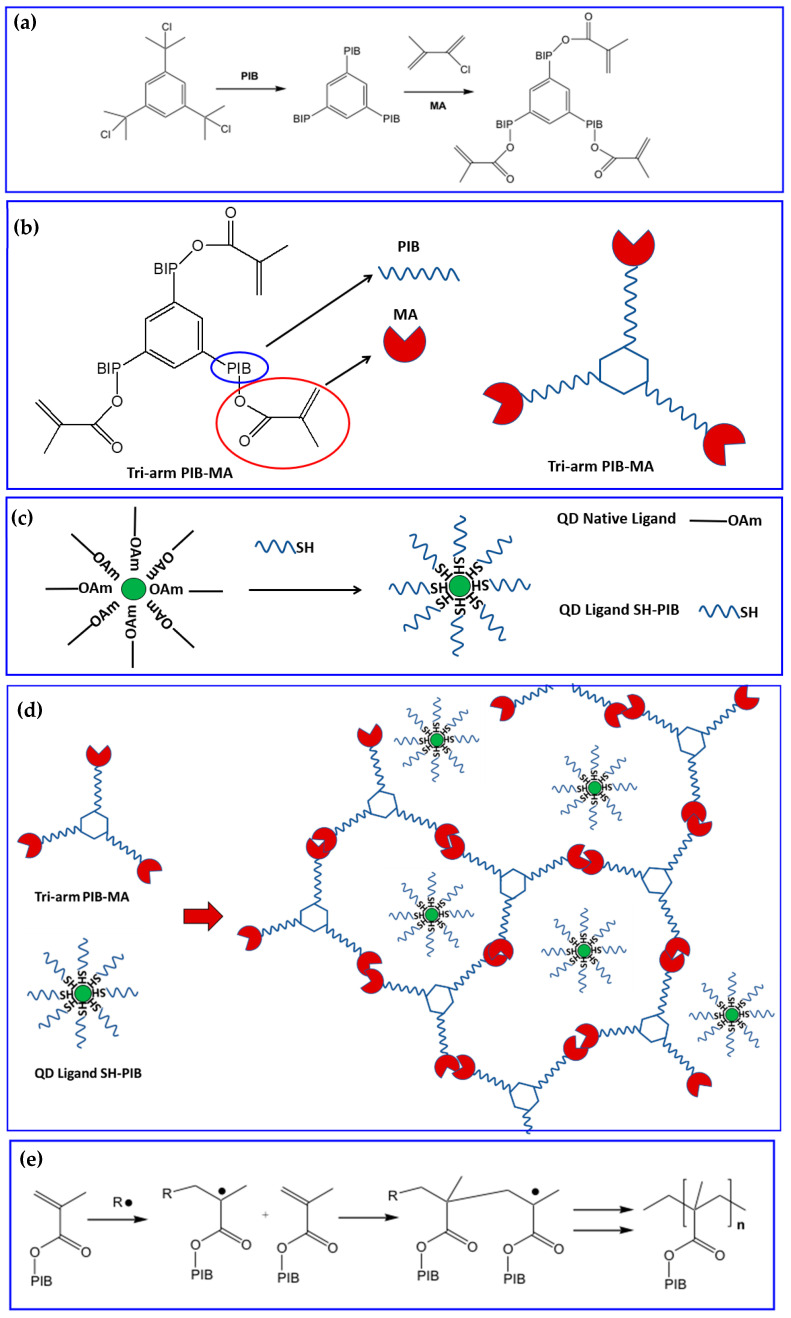
(**a**) Chemical reactions for the synthesis of the tri-arm PIB-MA with the reactive acrylate groups (MA); (**b**) scheme of the tri-arm star PIB-MA; (**c**) ligand exchange involving the native QDs where the native ligand is substituted by a ligand with a tail bearing the PIB group; (**d**) cross-linked network protecting the QDs; and (**e**) chemical reactions involving the methacrylate group activated by light.

The stabilization of the QDs was realized first by selecting a polymer, the polyisobuthylene (PIB), having specific properties like good flexibility, excellent oxygen and moisture barrier, thermal stability, optical transparency, and solvent resistance. Then, they modified the PIB by adding the cross-linking functions, the methacrylate (MA), which induce the formation of a covalent network [80] around the scQDs for their effective encapsulation. In particular, the PIB-MA reticulating polymer is obtained by linking the PIB to a “tri-arm star” aromatic center. Then, the PIB ends were functionalized with the methacrylate functions (Figure 14a,b) forming a three-arm star cross-linkable polymer (PIB-MA).

On the other side, the optimization of the scQDs’ dispersion is obtained through the exchange of the native scQDs’ ligand with a new ligand having as a spacer/tail the PIB oligomers. The ligand tail ensures the optimal interaction of the scQDs with the matrix, because they have the same chemical structure (Figure 14b,c), while the surface binding group, a thiol, ensures the strong binding of the ligand on the scQD surface during the ligand exchange procedure.

The cross-linking of the methacrylate units of the PIB [80] completes the protective action of the polymer around the scQDs (Figure 14d,e).

The optical characterization of the cross-linked film showed that the dispersion of the QDs was comparable with the one of similar polymeric films bearing the PIB without any crosslinking. Indeed, the photoluminescence emission and the PLQY are almost equivalent between these two types of samples. This effect suggests that the scQDs do not form any aggregates that quench the photoluminescence [41,42,43] and decreases the film transparency [40].

The stability tests were carried out in the air and vacuum under a high flux of light for 15 h, comparing the photoluminescence decay of the scQDs embedded in three different polymers: the PIB-MA, the SIBS, and the polylaurylmetacrilate (PLMA). These tests show that the cross-linked PIB-MA composite is more stable than the other type of composites, both in the vacuum and in the air, even if in the air the degradation is higher than in the vacuum.

Stability tests were also carried out by soaking the film in a HCl 5M solution for 35 days and the results showed that the scQDs enclosed in the cross-linked PIB hold their photoluminescence emission longer than the ones embedded in the other matrices.

#### 4.2.3. The Siloxanes

The siloxane organic–inorganic hybrid materials [81] (SHMs) are compounds that have the tetravalent silicon bonded with oxygen and one or more bonds replaced by a covalent linkage with an organic substituent [67,82]. With this kind of chemistry, the siloxane polymers bear ceramic-like properties joined with the ones of the organic materials. From the functional point of view, this fact means that these materials display both the ceramic “character”, i.e., high-temperature stability, hardness, chemical resistance, and optical transparency, combined with the organic “character”, i.e., low temperature and solution processing, modulation of matrix porosity, and flexibility.

The chemistry of the Si-O-Si bond was already illustrated in Section 3.1 (Figure 8), and here will be described the capacity of siloxanes to act as a collective encapsulating agent with high chemical thermal and mechanical stability, as recently presented by Bae’s group for the stabilization of scQDs in different conditions [83,84,85,86,87].

**Figure 15:**
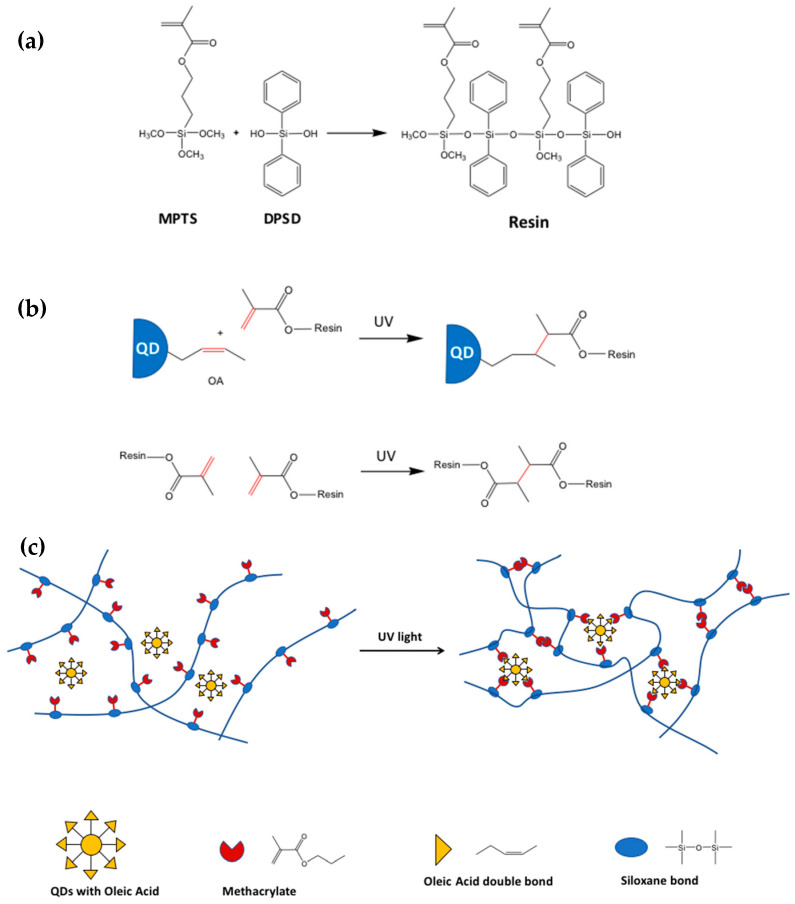
(**a**) Chemical reactions involved in the siloxane resin preparation, in particular, the sol-gel condensation of methacryloxypropyl-trimethoysilane (MPTS) with diphenylsilanediol (DPSD); (**b**) chemical reactions involving the formation of a covalent bond between the OA and the methacrylate group; (**c**) scheme of the siloxane network formation before and after crosslinking induced by light.

In the approach proposed by Bae’s group, the siloxane encapsulation method of scQDs consists of two steps (Figure 15): (i) the siloxane resin formation through the sol-gel method and (ii) the cross-linking of the resin with the QDs and with itself. In the first step is prepared the siloxane resin bearing the acrylate group by sol-gel condensation (Figure 15a). 

In the second step, the formed siloxane resin is mixed with the scQDs. The scQDs are usually passivated with oleic acid as a ligand that bears a double bond in its aliphatic chain.

The acrylate group can react both with the double bond of the scQDs’ ligand (the oleic acid) or with another acrylate moiety of the resin enhancing the resin networking (Figure 15b). The binding of the acrylate group with the double bond of the scQDs’ ligand or with another acrylate moiety is mediated by UV light after the addition of a photo-initiator like 2,2-dimethoxy-2-phenylacetophenone (DMPA) [88]. It is important to point out that the siloxane resin bearing the phenyl group of the diphenylsilanediol (DPSD) helps the enhanced dispersion of the scQDs in the resin through hydrophobic interactions [89].

In these conditions, the stability of the photon siloxane encapsulated scQDs (PSE-QDs) under thermal stress at 85 °C for 40 days and 85% relative humidity showed that normalized PL intensity remains constant during all of the period, as well as the PL lifetime. On the contrary, the scQDs simply mixed with the resin without cross-linking lost their photophysical properties, showing a 50% decrease in PL intensity and a strong decrease in the photoluminescent lifetime, with respect to the initial values.

**Figure 16:**
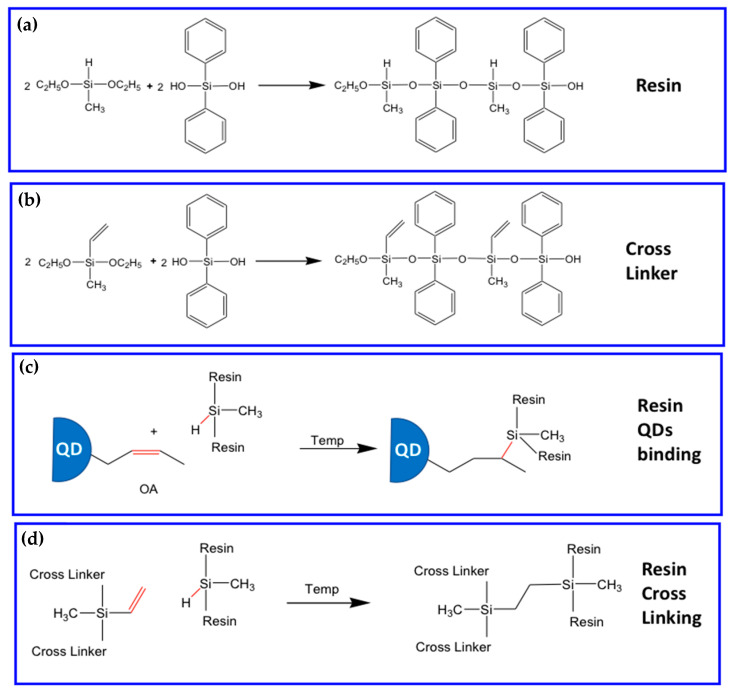
The thermally curable siloxane encapsulation strategy. The siloxane resin (**a**) and the siloxane crosslinker (**b**) bind the QDs (**c**) and the resin itself (**d**), respectively. In red are depicted the chemical bonds involved in the cross-linking formation between the resin and the scQDs’ ligand (oleic acid or oleylamine) and the siloxane polymers.

The siloxane thermal and environmental (oxygen and moisture) stability was improved by substituting the acrylate binding group with the vinyl group (Figure 16). 

Indeed, this siloxane resin, even if it is stable until 85°, cannot be enough for other applications like LED technology that requests temperatures above 100 °C. This phenomenon is mainly due to the organic acrylate moiety that decomposes at a high temperature.

In the new reaction scheme, the resin is substituted by two components: the resin bearing the reactive Si-H group and a linear cross-linker bearing the vinyl reactive group [85] (Figure 16a,b).

The vinyl group of the linear linker can bind the double bond of the oleic acid of the QDs blocking the scQDs within the resin (Figure 16c). In the meantime, the vinyl group of the linear vinyl organo-siloxane linker can bind also the Si-H bond of the resin, forming the network between the resin and the linear linker (Figure 16d). The thermal siloxane encapsulation (TSE) networking reaction in this case is stimulated by the temperature that is a curing step necessary for LED manufacturing. With this two-component matrix, the thermal stability of the networks has been improved until 180 °C, while the presence of the phenyl groups on the resin and cross-linker allows both the homogeneous distribution of the scQDs in the matrix [89] and a high refraction index (transparency).

The aging tests showed that the TSE-QDs are more stable at 120 °C 5% (30 days) relative humidity, with respect to the PSE-QDs, or simply mixed QD/acrylate (without any siloxane bonds). In the same way, the chemical stability was also tested for 30 days in four solvents (Ethanol, Acetone, HCl 0.1 M and, NaOH 0.1 M) that indicates that the PLQY remains stable at the initial value only for the TSE-QDs, with respect to the PSE-QDs and the ones mixed with the acrylate resin.

By using another type of siloxane resin bearing the epoxy siloxane group, it is possible to protect and include a micro-LEDs array suitable for biocompatible applications [83]. 

**Figure 17:**
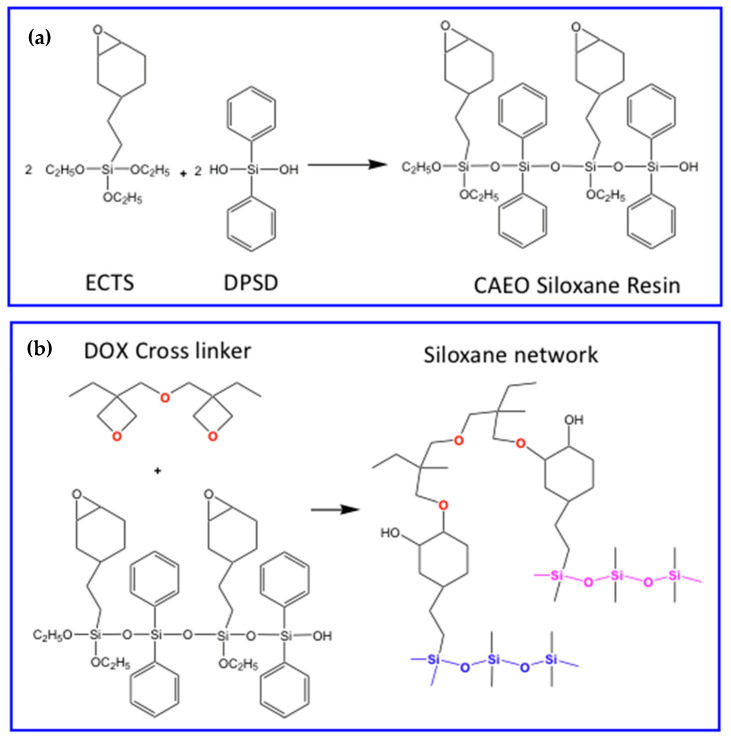
(**a**) Synthesis of biocompatible SHM starting from siloxane CAEO resin obtained with sol-gel method and (**b**) its cross-linking with DOX cross-linker activated with UV and temperature.

The siloxane matrix formation is similar to the previous ones and is prepared by adding the 2-(3,4-epoxycyclohexyl)ethyltrimethoxysilane (ECTS) with diphenylsilanediol (DPSD) via a sol-gel reaction to form the cycloaliphatic epoxy oligosiloxane (CAEO) (Figure 17a). This resin is cured with UV in association with bis [1-ethyl(3-oxetanyl)]methyl ether (DOX) acting as a cross-linker to form a siloxane network [86] (Figure 17b).

Once embedded in the siloxane matrix, the micro-LEDs array (f-HμLED) was characterized by studying its optical, thermal, thermomechanical, and environmental properties. Further biocompatibility tests were carried out on a similar device containing cadmium-based scQDs. The optical transparency was about 90% throughout the entire visible spectrum.

As an example, the study of the mechanical tests shows that the SHM exhibited the highest tensile modulus (6.8 GPa) with respect to those of polyethyleneterephthalate (PET) (4.8 GPa) and polyimide (PI) (3.8 GPa) films. On the other side, the SHM has the lowest flexural stress (2.5 GPa) than PI (3.9 GPa) and PET (4.8 GPa), indicating a high mechanical deformation including bending, folding, and twisting.

Another important test for the electronic device is its lifetime in the external surrounding conditions tested under 85 °C at 85% relative humidity (accelerated stress test). Here, the control matrix used for the device was the SU8 photoresist commonly used for patterning, and the variables examined are the device irradiance (ΔE) and the forward voltage (ΔV_f_). The behavior of both variables showed that after 15 days of treatment, the f-HμLED device is more stable than the μLED encapsulated with the SU-8.

The biocompatibility test was carried out with a similar device realized with cadmium-based scQDs embedded with siloxanes acting as color-converter films. The red and green Cd-based scQDs were mixed with the SHM resin, and the solution was poured over a GaN-based f-HμLED to obtain a white emitting source. This device was then used for biocompatibility tests for the presence of cadmium, and different type of cells were grown over this substrate and a control plate without the device. The results show that the growth rate of the cell lines grown over the device versus the same cell lines grown in the control plate are comparable within the statistical level of significance, validating the biocompatibility of the material.

## 5. Quantum Dots Direct Optical Patterning (DOP)

The research on the patterning technologies of scQDs is an active area of study for their industrial application due to the high interest of companies, especially in display manufacturing [62,90]. Photolithography is the most widely used technique in the industrial field; however, the use of photoresists and the multiple steps of etching/washing can alter dramatically the QDs’ functionality and the production costs, respectively.

Recently, different authors published some works that utilize direct optical patterning (DOP) as a step forward in photolithography. Indeed, the use of the light for direct patterning associated with smarter chemical production of the materials can ensure stability, dispersion, and a relatively simple patterning process of scQDs. Another advantage of DOP is that it can utilize the same equipment used for the photolithography so that only minor changes would be necessary in the production chain, minimizing the upgrade costs.

In the following paragraphs are presented five different chemical approaches that use the same patterning methodology, the DOP, but different chemical processes to tackle the issues of stability, dispersion, and patterning itself. The purpose of this comparison is to produce a starting point for evaluating the pros and cons of the various proposed techniques.

### 5.1. Direct Optical Patterning of scQDs with Thiol-Ene Cross-Linkers

In Section 4.2.1 the effect of the thiolene encapsulation on scQDs stability was already reported. However, the same chemical process can be adopted for the photolithographic patterning of scQDs as shown by Li et al. [91].

The authors prepared a solution containing the tri-branched molecule (TAIC) bearing the double bond necessary for the thiol-ene reaction with the four-branched thiol (PTMP) and the scQDs. This solution was directly drop-casted over a blue LED and then irradiated with a UV LED focused with an objective lens on a moving stage to carry out the direct optical patterning. The authors using this equipment obtained a patterning resolution between 6 μm to 40 μm. They also compared the optical characteristics of the crosslinked film with the solution and with a drop-casted film. In general, they observe that the PLQY, the FWHM, and the wavelength shift of the patterned film are better than the ones of the drop-casted film. This fact suggests that the cross-linking help the scQDs’ dispersion. On the other side, the solution shows better optical performances, because the chemical characteristics of the film were not optimized.

**Figure 18:**
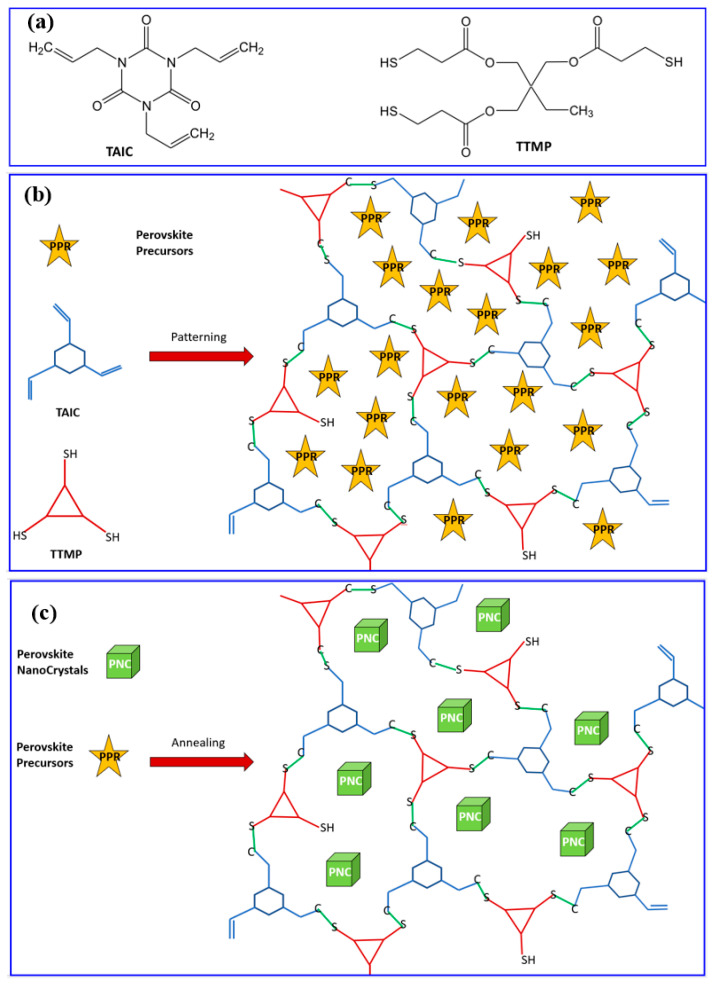
(**a**) Chemical reagents to form the thiolene network; (**b**) the formed thiolene network including the Perovskite precursors (stars); (**c**) the annealing process that induces the perovskite nanocrystals formation (cubes).

Zhang et al. [92] made a significant improvement patterning the perovskite scQDs. The main novelty of this work is that the synthesis of the perovskite scQDs is realized after the thiol-ene network formation. Indeed, first, the thiol-ene matrix is formed, and then the perovskite nanocrystals are grown through the annealing of specific perovskite precursors trapped in the matrix. In particular, the process of formation can be described as four steps (Figure 18): (i) all the precursors for the formation of the encapsulating network (TAIC and TTMP) and the of the perovskite are dissolved together by using N,N-dimethylformamide (DMF) and dimethylsulfoxide (DMSO) solvents; (ii) this ink is then used for film deposition, and patterning with UV light that “freezes” the perovskite precursors in the patterned positions; (iii) the un-patterned areas are removed by washing them; (iv) the perovskite nanocrystals are then grown with an annealing process.

The effect of this strategy is that the final perovskite scQDs film is homogeneous because the precursors are all soluble in the prepared solution; hence, the patterned areas are also homogeneous as luminescence emission. According to the authors, the growth of the perovskite after the UV patterning avoids any perovskite damage due to UV treatment.

The patterning resolution tests show the possibility reaching a limit of 5 μm, depending upon the degree of resolution of the mask.

The PLQY stability of the scQDs within the film after 30 days at 54% humidity and ambient condition can retain 85% of its initial value. Similarly, the PL signal remains constant for 30 days under UV light, confirming the stability ensured by the encapsulation. A further test of protection was carried out by immersing a patterned film in water and ethanol, demonstrating that after 10 h of immersion, the PLQY drops down to 66% and 60% of its initial value, respectively.

A limiting factor of this work is that, to add more layers to enhance the film thickness, it is necessary to cover the underlying patterned layer with another film of SiO_2_ to protect the lower layer from the attack of the washing solvents (DMF and DMSO).

### 5.2. Direct Optical Patterning of scQDs with Siloxanes

The possibility using the DOP combined with the siloxane chemistry to pattern and protect the scQDs has been readily demonstrated by Bae’s group for the manufacturing of quantum dots color filters (QD-CF) for displays [31]. In this work, the authors solved three main issues encountered when building color filters, namely: (i) dispersion of high loading of QDs (>20 wt %), (ii) stability against heat and moisture; and (iii) photo-patternability.

**Figure 19:**
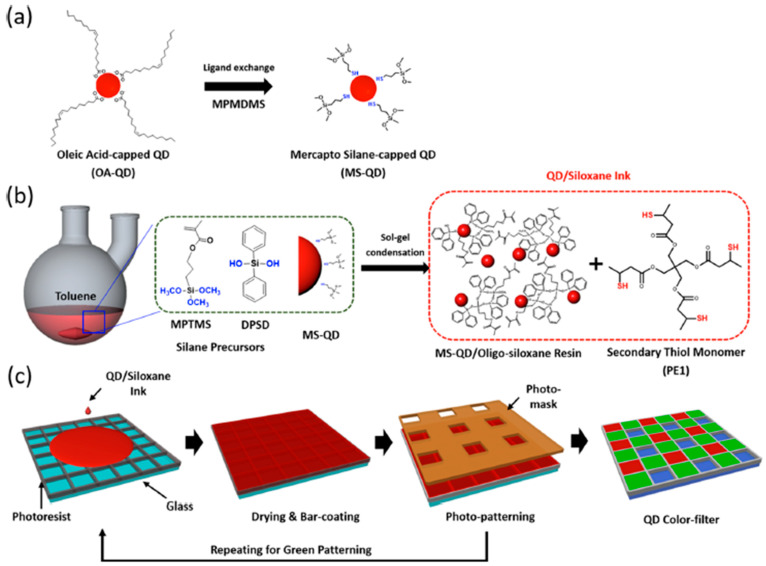
The three steps of quantum dots color filters realization starting from (**a**) QD ligand exchange; (**b**) preparation of the QDs/siloxane ink by mixing the silane monomers and the thiol monomer (PE1) for the thiol-ene chemistry; and (**c**) the photo-patterning process over the glass where an array of squares was pre-patterned. Then, the QD/siloxane ink is deposited and patterned with DOP (Reprinted with permission from [31]. Copyright © 2022 American Chemical Society).

The high loading of QDs poses a problem for scQDs’ dispersion within the siloxane matrix that was bypassed by exchanging the native scQDs’ ligand, the oleic acid, with a mercaptopropyl-methyl-dimethoxy silane (MPMDMS) (Figure 19a). The tail of this ligand bears the dimethoxy groups that can react with the hydroxyl groups of diphenylsilanediol (DSPD) during the siloxane resin formation (sol-gel condensation) (Figure 19b). The presence of diphenyl rings performs a dual function, i.e., (i) it ensures a good scQD dispersion, preventing further aggregation during the next steps of the patterning; and (ii) it ensures also a good optical transparency of the final film. The resin bears another important function, through its methacrylate group, introduced by the 3-methacryloxypropyltrimethoxysilane (MPTMS). This group is, indeed, necessary for the binding with the thiol cross-linker that allows the encapsulation/patterning.

The siloxane solution (ink) for the encapsulation/patterning was prepared by adding to the siloxane resin a four-branch thiol (PE1) that deals as a reticulating agent coupling with the methacrylate group of the siloxane resin (Figure 19b).

The photo-patterning (Figure 19c) was performed by pouring the siloxane ink (with red scQDs) into a pre-patterned array (formed by 50 μm × 50 μm squares). The reaction of the thiol PE1 with the acrylate functional group is a typical process of the thiol-ene chemistry [93] and is activated by light. The same procedure is, then, repeated by using the green scQDs.

It is important to highlight the high loading of QDs used for the experiments was 10 wt % for red scQDs and 20 wt % for green QDs, and with this loading the film produced is homogenous.

The stability tests were carried out in conditions of high temperature and humidity, namely at 85 °C and 5% and 85% of moisture, in comparison with a film realized with a standard photoresist. In these conditions, the PLQY and the time decay were stable as the initial value for the QDs encapsulated with the siloxane, while the stability of the scQDs embedded in the standard photoresist drops down after one day and it is almost 0 after 30 days. This fact further confirms that the siloxane encapsulation is effective and ensures also an optimal dispersion.

The chemical stability was also verified in ethanol and strong acid and base for both encapsulants, i.e., siloxane and photoresist, for 30 days. The test was successfully surpassed only by the siloxane encapsulant in which the PLQY of the scQDs remains stable as the initial value.

It is worth mentioning the work of Ozdemir et al. [94], because they use siloxane chemistry to protect each single QD that is then patterned by using a commercial photo-patternable resin. In particular, the CdSe/CdS scQDs are, first, encapsulated in a silica shell that is then functionalized with methylacrylate ligand to improve its dispersion in the photo-patternable resin. In such a way, each single scQD is protected from oxygen and moisture while the acrylate function ensures the necessary dispersion in the photo-patternable resin. The mixture formed by CdSe/CdS@SiO_2_ and resin is the solution that is deposited and patterned using light.

### 5.3. Direct Optical Patterning via In Situ Ligand Exchange (DOLFIN)

**Figure 20:**
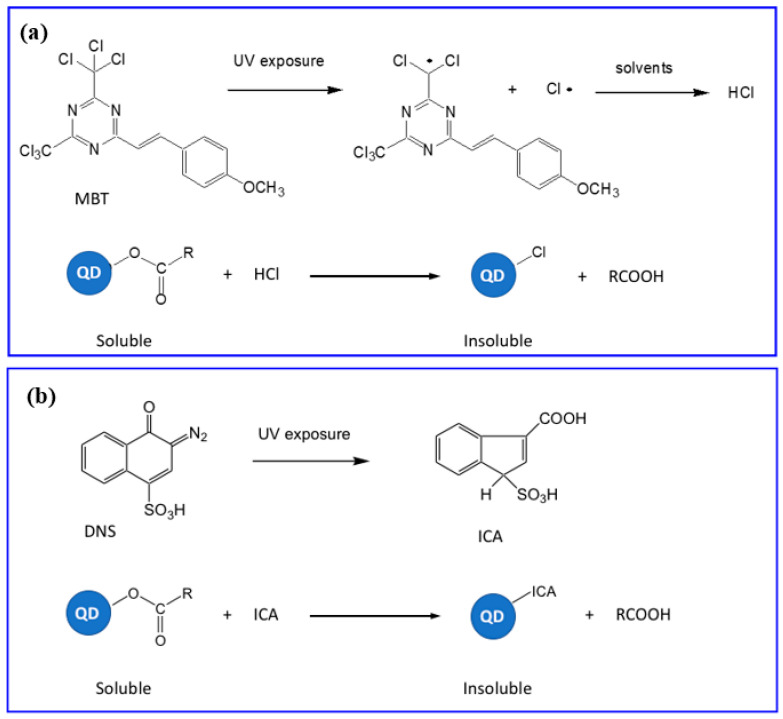
(**a**) The MBT after irradiation and in presence of solvents produces HCl that remove the carboxylate from the QD surface that changes its solubility (it precipitates); (**b**) the DNS upon irradiation became ICA that substitutes the carboxylate on the surface inducing the QDs’ precipitation.

The patterning strategy that uses the ligands of the scQDs as active molecules for the patterning itself was proposed by Talapin’s group [95,96] (direct optical lithography of functional inorganic nanomaterials DOLFIN). This is the first example of patterning in which the matrix is absent while the photoresist is a constant of the photolithography. This approach is particularly elegant because it forecasts the exchange of the scQDs’ ligand “in situ”. The effect of the ligand exchange is the precipitation (change in solubility) of the patterned QDs. The key molecules of this process are the so-called photoacid generators (PAGs) that have to be combined with the scQDs in special ink. Figure 20 illustrates the PAGs’ action. The 2-(4-methoxystyryl)-4,6-bis(trichloromethyl)-1,3,5-triazine (MBT), for example, after UV exposure produces chlorine radicals that in presence of solvents (toluene, acetonitrile) induce the formation of HCl that protonates and detaches the oleic acid from the scQD surface. In these conditions the scQDs become insoluble and precipitate (Figure 20a).

Similarly, the 2-diazo-1-naphthol-4-sulfonic acid (DNS) after the UV exposure produces a sulfonated derivative of the diazonaphtoquinone (ICA) that substitutes the oleic acid at the scQDs surface, which becomes insoluble (Figure 20b). The complete path of patterning can be resumed in four steps as reported in the following. The PAGs molecules and the scQDs are mixed forming the so-called photo-patternable emissive nanocrystals (PEN) ink, the ink is deposited over the substrate forming a film, the film is then irradiated, and the film is developed using a non-polar solvent that washes away the unexposed soluble scQDs. The selection of the PAG molecules is particularly important because they have to be soluble in the preparation. The patterning methodology has been tested in terms of resolution to reach up to 1.5 µm.

Talapin’s group tested also the effect of this patterning methodology over a QD-LED, showing that the electro-optical characteristics of the device are almost identical to a similar device realized with pristine scQDs.

**Figure 21:**
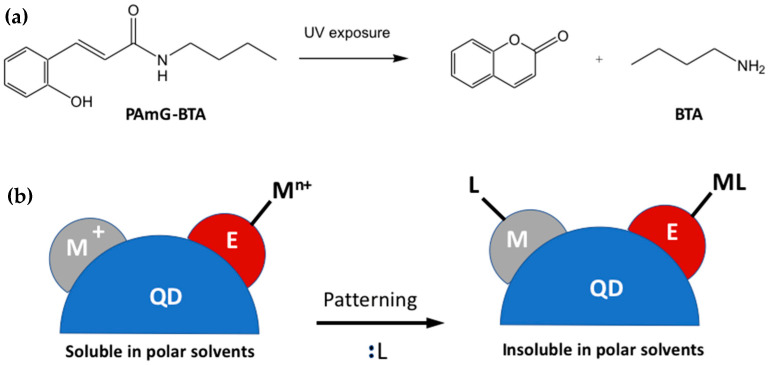
(**a**) A condensed form of the cumaric acid with butylamine (PAmG-BTA) under UV light produces the butylamine (L-type ligand, 2 electron donor); (**b**) the butylamine complexes the metal atoms (M) both at the QD surface and the one bonded to the chalcogenide site (E).

Recently, Talapin’s group also demonstrated the same approach, the change in the QDs’ solubility through the in situ modification of the ligands, by using intensely luminescent all-inorganic nanocrystals (ILANs) [12] for the direct optical patterning. In this work, the ILANs were combined with a molecule that, after irradiation, produces a type L ligand that complexes the surface of the ILANs themselves, changing their solubility. In particular, the ILANs were combined with a PAmG-BTA molecule and then deposited. The PAmG-BTA has the property that, decomposes after UV exposure, giving the n-butylamine (BTA) (Figure 21a). The butylamine is an L-type ligand (two-electron donor) that form complexes with the exposed metal sites at the scQDs surface. This change in ligand switches the solubility of the scQDs in polar solvent so it is possible to obtain patterned areas (Figure 21b).

The resolution of the patterning arrives up to 2 µm, and the thickness of the layer can be modulated by controlling the spin coating parameters and the density of the solutions. The limitation of this work is the decrease in the PLQY always associated with the inorganic ligand exchange; indeed, the PLQY of the red scads decreases from 80% (un-patterned) to 75% (patterned), from 76% (un-patterned) to 68% (patterned) for green scQDs, and from 78% (un-patterned) to 58% (patterned) for the blue-emitting scQDs.

### 5.4. Direct Photolithography of scQDs via Photo-Active Cross-Linkers

Another DOP strategy in which the photoresist/matrix is absent is the one using the azide cross-linkers [32,97,98,99]. The approach is similar to the one explored with the polymers and siloxanes but uses only a cross-linker molecule while the matrix is absent. The role of the cross-linker activated by light is to create a network between the scQDs through their organic ligands. With this methodology, all the steps that require the scQDs manipulation, like the ligand exchange or solvent change, or other chemical manipulation necessary for the deposition and patterning of the scQDs, are avoided or limited. In particular, the patterning strategy proposed by the Kang group uses a bis-Per Fluoro Phenyl Azide cross-linker (bis-PFPA) that can bind two different carbon atoms of the perovskite QDs ligands after light activation [97,98].

**Figure 22:**
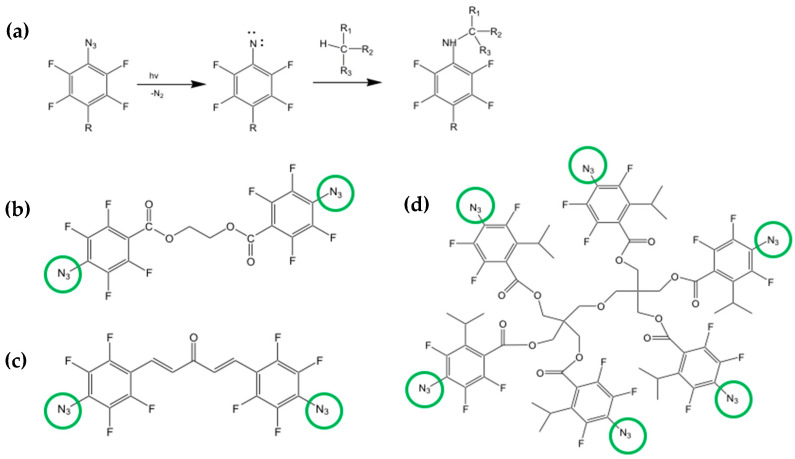
(**a**) Mechanism of nitrene (green circle) activation with UV within the PFPA molecule; (**b**,**c**) two different types of bis-PFPA used to cross-link at different wavelengths; (**d**) six-arm PFPA with isopropyl groups to increase the steric hindrance improving the quality of photo-patterning.

Figure 22a shows the chemistry of the PFPA. The active group of the molecule is the azide (N_3_) that, after the activation with light, binds a quaternary carbon with a carbon-nitrogen bond [100].

The bis-PFPA are bifunctional cross-linkers bearing two azide groups in opposite positions (Figure 22b,c) that can act as a bridge between two different carbon atoms.

**Figure 23:**
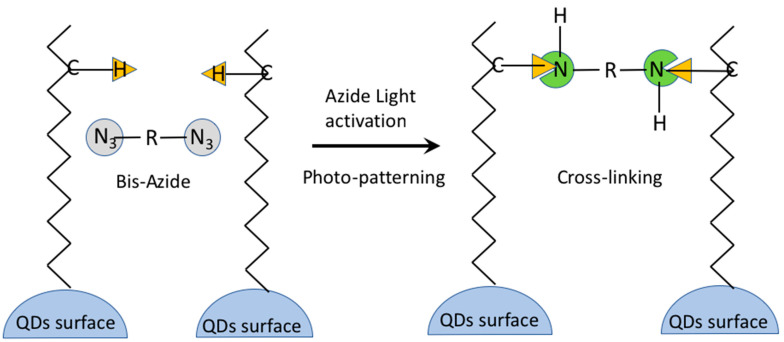
Scheme of cross-linking between different ligands of scQDs realized through bis-azide linker. The drawing illustrates the binding between the nitrogen of the crosslinker and the carbon atom of the ligand. The bis-azide acts as a bridge between two different ligands of two neighboring scQDs.

When the bifunctional cross-linker is mixed with the scQDs, it reacts with two different carbon groups of two aliphatic ligand chains of the neighboring scQDs (Figure 23). A careful evaluation of the cross-linker amount is necessary because it can damage the QDs’ optical properties. However, this drawback has been solved by the same research group by modifying the structure of the PFPA by introducing a multiple-arm PFPA (Figure 22d) [99]. Indeed, the bulky structure of the multiple-arm PFPA prevents the photoactivated nitrene radicals to join the scQD surface, compromising the PLQY.

With the use of a six-arm PFPA, the final pixel diameter obtained with the photo-patterning is 6 µm, 4 µm, and 2.5 µm. This methodology was utilized to manufacture an electron-driven QD-LED as a test for the effect of the cross-linker and the device stability in comparison with a QD-LED without any cross-linker [99]. In both devices, the current-voltage characteristics are almost identical; however, the time at which the luminance became 90% of the initial one (L = 1000 cd m^−2^) is 156 h for the cross-linked device with respect to 15 h of the QD-LED without cross-linking. The authors suggest that the increased robustness of the device is a consequence of the scQDs cross-linking that decreases the interfacial defects between the scQDs layer and the ZnMgO electron transporting layer.

**Figure 24:**
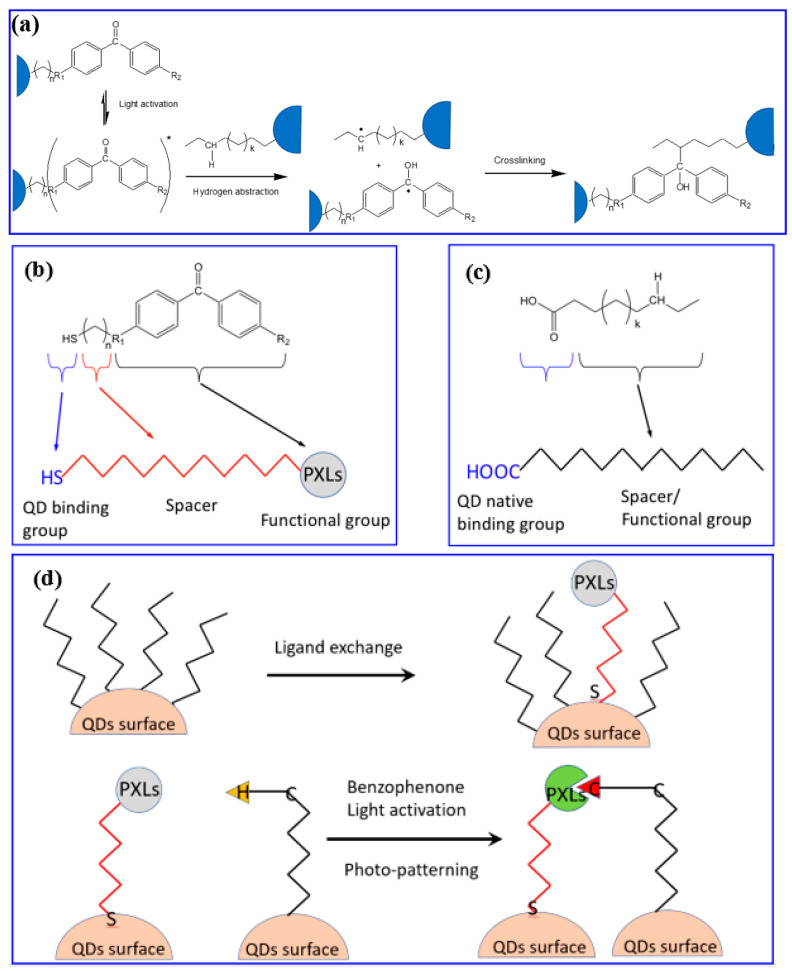
(**a**) The chemical reactions involved in the benzophenone cross-linking; (**b**) the structure of the benzophenone-modified ligand bearing a thiol group that displaces (**c**), the native carboxylate ligand; and (**d**) the drawing of the steps leading to the photo-patterning with benzophenone: top, the ligand exchange and down, the formation of the cross-linkage.

Progress with the cross-linkers was made by introducing a benzophenone-derived ligand for direct optical patterning [32]. This strategy introduces the benzophenone cross-linker as an end group of the scQDs’ ligand instead of adding an external molecule like the bis-azides. Of course, this new ligand has to be exchanged with the native ligand of the synthesized scQDs. The benzophenone acts as the azide cross-linker by binding a four-valence carbon group of a neighboring ligand of another scQD (Figure 24a). The patterning strategy starts with the QDs’ ligand exchange to replace some of the native ligands with the benzophenone-modified ligand (Figure 24b–d). Then, the light activates the process of cross-linking, and patterning (Figure 24d).

The advantages presented by this strategy are based on the synthesis of a ligand bearing as end group the benzophenone and as a surface binding group a thiol. This new ligand is exchanged with the native ligand and only a small amount of substitution (below 10%) is necessary to have a good cross-linking. The final scQDs are a two-ligand system that allows an optimal scQD dispersion within each type of solvent. Indeed, one ligand ensures the cross-linking while the other one the solubility. And this is an important factor that tackles also the issue of the solubility of the cross-linkers with the solvents of the scQDs and allows the use of these solutions also for ink-jet patterning. Another advantage with respect to the bis-azide cross-linkers is that the latter can hinder the charge transport between the QDs.

The authors tested both the optical properties of the patterned scQDs and the patterning resolution. The PLQY of the ligand-exchanged scQDs remains unchanged after the ligand displacement due to the mild exchanging procedure and even after the cross-linking reaction. The patterning resolution was high indeed; the authors were able to reach patterns with widths from 3.8 µm up to 0.8 µm. Another interesting feature of the chemistry of the patterning is that it can be repeated layer-by-layer without any buffering coating.

The authors also tested the patterned scQDs in an electron-driven QD-LED and all of the electrical and optical characteristics are almost identical to the ones using the pristine scQDs.

### 5.5. Direct Optical Patterning of scQDs via Their Direct Synthesis

The direct synthesis of the scQDs using a laser within a film [101] can be considered belonging to the DOP strategies. The main technical difference with respect to the previous methodologies is that the laser was used to directly synthesize the scQDs.

**Figure 25:**
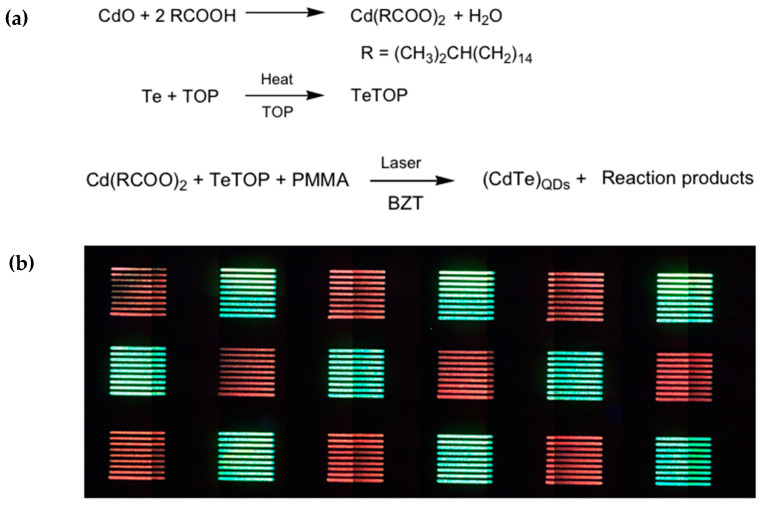
(**a**) Synthesis of the cadmium and tellurium precursors, the cadmium carboxylate and TOP-Te, and their chemical reaction during the laser synthesis within the PMMA layer; (**b**) red and green squares formed by CdTe QDs are obtained modulating the laser pulse frequency, dose, and speed. The size of the squares is 1 mm and the image were obtained with the fluorescence microscope exciting the film with UV radiation (Reproduced with permission from [102]).

This technique, often called direct laser patterning [101], combines the flexibility of the laser technique both at the industrial and technological level with the chemistry and optical properties of the QDs. Indeed, recently it was demonstrated that by changing the laser parameters, like the pulse power and frequency, it is possible to pattern (to grow) the scQDs, modulating their optical properties [102]. This kind of result has been possible by designing the chemical precursors within the film to be patterned in a proper way. This means including in the chemical formulation the suitable scQDs precursors, polymeric matrix, and other chemicals, allowing the laser-stimulated scQDs’ growth (Figure 25a).

Among these components, it is worth mentioning the BZT, (2-(2H-Benzotriazol-2-yl)-4,6-ditertpentylphenol), which is a molecule that absorbs the laser light converting it into thermal energy, that is the driving force of the QDs’ synthesis.

On the other side, equally importantly, are the laser parameters that condition the achievement of the correct optical properties of the scQDs. Indeed, given a film chemical formulation, only specific combinations of pulse frequency (laser repetition rate), laser power, and beam speed allow to reach the proper conditions to grow the green- or red-emitting CdTe scQDs regions (Figure 25b).

The heat generated by BZT after the absorption of the UV laser radiation induces the formation of the CdTe scQDs. Indeed, the CdTe precursors were designed to form the CdTe scQDs by a thermal process when incorporated in a similar polymeric matrix [103]. By observing the parameters necessary to obtain red or green crystals, it was evident that the laser dose and the laser repetition rate are the key factors determining the optical properties of the CdTe scQDs. In particular, the pulse frequency is the key factor to obtain the red and green squares, because the pulse frequency is correlated with the temperature of the film. Indeed, it was shown that an annealing process carried out at relatively low temperatures favors the green scQDs, while at higher temperatures the growth of the red scQDs is stimulated [103].

The laser pulse frequency influences the film temperature, because at 20 kHz, for example, each laser pulse is separated from the next one by 50 μs and this means that the heating and cooling steps are separated enough to achieve the correct temperature of the matrix suitable to grow the green-emitting scQDs. At higher frequencies (80–100 kHz), the time distance from the pulses is in the range 1–10 μs, and the film has not enough time to cool down. This means that the temperature of the film is higher enough to induce the red-emitting scQDs’ formation.

The limitation of this work is about the scQDs’ stability. Indeed, the PMMA matrix does not protect the CdTe scQDs from environmental conditions (oxygen and moisture), so the PL intensity and the time stability are poor. However, this work demonstrates that direct optical patterning can be a suitable strategy to modulate the QDs’ optical properties and that the use of a laser can replace the masks’ manufacturing maintaining a high resolution and automation typical of lasers.

## 6. Conclusions

This report has shown a path that goes from the properties of the scQDs to their patterning, passing through the methods for improving their dispersion and stability in a matrix. Direct optical patterning (DOP) is an emerging tool to simplify the patterning process of the scQDs for display manufacturing that, however, is strictly related to the chemical processes that lead to the stability and homogeneity (dispersion) of the QDs for the correct function of the device.

Stability and dispersion of the scQDs impact several fields from displays, photovoltaics, photodetectors, bioimaging, medicine, and many other fields as reported in the recent literature [4,104]. This report showed many examples of scQDs’ exploitation in different areas. Bioimaging exploits the stability and wide gamut of the emission color coupled with the possibility being bound to protein as markers for cell imaging [105] and for immunoassays as reported by Goryacheva [69].

Photovoltaics is another area of expansion for the applications of scQDs. Indeed, specific core/shell systems reached a quantum efficiency of 1 [20]. The core/shell structure of the scQDs can be designed to enhance their stability and to optimize their spectral properties, like in the case of the giant scQDs [21,22].

Photodetectors are devices converting incident photons to an electrical signal and PbS scQDs embedded in a polymer are used as sensors for IR radiation. These sensors are very appealing for their easy fabrication in solution, compatibility with different substrates, and broad sensitivity [106].

A lot of work is carried out for the inclusion of the scQDs in displays [107,108] and many efforts were summarized in this review showing how different authors overcome and combined the issues of the stability, dispersion, and patterning of the scQDs.

The optimization of the dispersion of the scQDs within a matrix is the golden rule to homogenize the interaction of the scQDs with the matrix itself. This means that the organic ligand at the QD surface should have the same chemical nature as the matrix.

On the other side, stability is ensured by the formation of a close network of covalent bonds that cages the scQDs, preventing the loss of the ligands and surface atoms and, hence, preserving their optical properties.

Considering these two main boundary conditions, five different approaches of DOP that exploit different chemical processes are evaluated. Even if often a comparison is complex, due to the different tests carried out in different types of samples, some general evaluation can be carried out.

The thiol-ene approach to DOP uses two simple multi-branched molecules, one thiol and one with a double bond, to encapsulate and pattern the scQDs [91,92]. The limitation of these works lies in the fact that the encapsulating network is still not designed to improve the interaction between the network itself and the QDs. In this sense, the work presented by Lesnyak’s group with the PIB polymer is more suitable [79]. Indeed, the PIB cross-linkable polymer and the scQDs functionalized with PIB-ligand improve the dispersion and stability of the QDs in the matrix. Even if this approach was not been tested for patterning, the fact that, with the help of light, the polymeric network is formed means that the DOP can reasonably be applied.

The DOP associated with the chemistry of the cross-linkers, like azides [109] or benzophenone [32], is one of the matrix-free methods. This method requests a moderate impact in chemistry for the synthesis of specific azides or ligands bearing the benzophenone end group. Once the impact of the chemical manipulation has been overcome and this should be taken into consideration for an industrial application, the patterning is relatively simple. The patterning approach of Talapin’s group [95,96] is an elegant way to control the position of the QDs, but the ligand exchange presents a decrease, even if modest, in the PLQY that influences the efficiency of the whole system. In these matrix-free methods, the absence of an encapsulating agent does not ensure the stability of the material over time and in harsh conditions, even if it is possible to conceive that the presence of a matrix should not compromise the patterning strategy, as also reported by Hahm et al. [32].

The direct synthesis of the scQDs by laser [102] is interesting from the scientific point of view. However, without the setup of an encapsulating agent it is not suitable for market exploitation.

In all of the presented works, the research teams reported the manufacturing of patterns with the DOP; however, only some of them realized a working device to show the feasibility of the process (Table 1).

In general, all of the techniques showed the possibility to obtain patterns even with high resolution for pixel manufacturing (below 10 μm or even less). Only in three cases the devices were successfully realized. The main difference in these works is the evaluation of the device’s stability. As stated above, the functional tests of the realized devices are quite different and only Bae’s group carried out a test in harsh conditions (high humidity for a long time and in the presence of acids/bases).

The patterning strategy using the siloxanes [31] seems to be the most mature approach because it was developed over time to surpass dispersion, moisture, temperature, mechanical and biocompatibility tests. Bae’s group, indeed, “played” with siloxane chemistry introducing time by time different chemical groups to improve the dispersion of the scQDs, the optical transparency of the matrix [89], the stability at higher temperatures [85], or the mechanical performances of the siloxanes [83]. In addition, the chemistry of the siloxanes is well-developed and almost all the reagents described are commercially available.

In conclusion, the combination of the use of the laser for DOP with the mentioned chemical strategies starting from the chemistry of the siloxanes could be an interesting benchmark to find the best chemical path for the patterning of functional scQDs. Indeed, what is appealing in the use of the laser as patterning equipment is that (i) it is not necessary to have a mask for patterning, (ii) the laser is easily driven by a PC (beam position and energy), (iii) it maintains a high resolution [110,111], and finally, (iv) from an industrial point of view, it is a mature technology with a broad industrial penetration.

Finally, DOP can bring significant cost savings in display manufacturing. Indeed, it is not necessary to pay for the preparation of the masks or the washing/curing steps typical of the lithographic process. Furthermore, there should be no additional investment costs as DOP can be conducted with existing equipment. On the other side, the introduction of DOP increases the impact of the chemical reagents for dispersion, protection and, patterning of scQDs. Only a good balance between the process simplification and the impact of the chemistry evaluated together with the final performance of the device can bring to a real market application.

## Figures and Tables

**Table 1:** Feasibility of the process.

DOP Technology	Patterning Resolution (µm)	Device Realized	Device Stability
Thiol-ene	5 [92]	n.d.	n.d.
Siloxanes	10 [84]	[31]	30 days 85% Humidity Harsh conditions
DOLFIN	1.5 [96]	[96]	Comparison with standard device
Cross-linkers	2.5 [99]	[99]	Comparison with standard device
Laser	30 [102]	n.d.	n.d.

## Data Availability

Not applicable.
